# Dimerization in tailoring uptake efficacy of the HSV-1 derived membranotropic peptide gH625

**DOI:** 10.1038/s41598-017-09001-x

**Published:** 2017-08-25

**Authors:** Annarita Falanga, Salvatore Valiante, Emilia Galdiero, Gianluigi Franci, Olga Scudiero, Giancarlo Morelli, Stefania Galdiero

**Affiliations:** 1 Department of Pharmacy and CiRPEB- University of Naples “Federico II”, Via Mezzocannone 16, 80134 Napoli, Italy; 2 Department of Biology, University of Naples “Federico II”– Monte Sant’Angelo, 80126 Napoli, Italy; 3 National Institute of Biostructures and Biosystems (INBB), V.le Medaglie d’Oro, 00136 Rome, Italy; 4 Department of Experimental Medicine, Università degli Studi della Campania Luigi Vanvitelli, Via Costantinopoli 16, 80138 Napoli, Italy; 5 Department of Molecular Biology and Medical Biotechnology, University of Naples “Federico II” 80131 Napoli, Italy and CEINGE-Biotecnologie Avanzate Scarl, Via G. Salvatore 486, 80145 Napoli, Italy

## Abstract

gH625 constitutes a promising delivery vehicle for the transport of therapeutic biomacromolecules across membrane barriers. We report an application of multivalency to create a complex nanosystem for delivery and to elucidate the mechanism of peptide-lipid bilayer interactions. Multivalency may offer a route to enhance gH625 cellular uptake as demonstrated by results obtained on dimers of gH625 by fluorescence spectroscopy, circular dichroism, and surface plasmon resonance. Moreover, using both phase contrast and light sheet fluorescence microscopy we were able to characterize and visualize for the first time the fusion of giant unilamellar vesicles caused by a membranotropic peptide.

## Introduction

The plasma membrane plays numerous functions in maintaining cell survival but it also represents one of the major impediments for the delivery of therapeutic agents into cells, the main limitation being the poor passive cell membrane permeability of hydrophilic molecules/drugs. Thus, enhanced delivery across cell membranes provides an outstanding potential for the treatment of human diseases and for the study of cellular processes and functions, as demonstrated by the great interest aroused in the development of delivery approaches. A possible strategy to overcome the membrane barrier is represented by the use of basic peptide sequences, the so-called cell-penetrating peptides (CPPs), with the ability to autonomously cross biological membranes without the assistance of cell membrane receptors and without cell rupture thus in a nontoxic and non-immunogenic manner^1^. This provides a good strategy to deliver a large variety of cargo molecules for therapy and diagnosis granting a substantial improvement in the cellular uptake. Despite the debates concerning the uptake mechanism, there seems to be a consensus that more than one mechanism may be involved. Nevertheless, a wider application of CPPs in cellular delivery has been hampered by their entrapment in cellular organelles, which seriously reduces the cargo delivering efficacy; for this reason, a physically driven mechanism appears to be a more efficient process for the delivery of macromolecules of biological relevance. To this purpose, one of the main challenges of recent research is the obtainment of novel delivery systems that are able to cross membranes using different internalization mechanisms without being entrapped in endosomes^2^.

Membranotropic peptides have gained tremendous interest because of their outstanding potential for the delivery of macromolecules across cell membranes offering great opportunity for the treatment of human diseases^2, 3^. The key for their ability to function as vectors is the membrane distortion caused by their partitioning at the membrane interface; thereby, membranotropic peptides modify the biophysical properties of phospholipid membranes; nonetheless, the exact mechanisms of insertion into and modification of target cell membranes are still unknown^1–4^.

The pioneering membranotropic peptide for enhancing delivery across membrane bilayers is the twenty residue peptide gH625, which was identified in our laboratory^5^. It belongs to the glycoprotein H of *Herpes simplex virus* type I (HSV-1), and is a membrane-perturbing domain playing a part in the merging of the viral and cellular membranes^6–8^. The peptide contains residues that are critical for the interaction and destabilization of the target lipid membranes; the amphipathic α-helical conformation attained in membranes, plays a major role in mediating lipid-protein interactions during the binding to membranes^9^. Once bound, its amphipathic nature would endow gH625 with the capability of entering membrane interior with an oblique orientation, thereby triggering local fusion of the membrane leaflets, temporary pore formation, cracks, and membrane fusion^9, 10^.

gH625 has shown exceptional vector properties and, in fact, it is able to efficiently deliver a range of cargoes producing the desired biochemical effect in the expected cellular localizations^5^. In addition to the remarkable efficiency rates, gH625 is essentially internalized by a non-endocytic pathway avoiding endosomal entrapment^10–20^ and is able to penetrate the Blood Brain Barrier both *in vitro* and *in vivo* ^11, 20–22^. gH625 is therefore a very good delivery vector and a complete understanding of the uptake mechanism and molecular details of its membrane interactions are of major relevance for optimization of this and similar delivery tools.

We have previously demonstrated a strong correlation between the affinity of gH625 for the lipid bilayer and cellular delivery efficiency, which is consistent with its uptake by a physically mediated process^23–26^; in fact, to fully describe the biophysical mechanism involved in the molecular mode of action of gH625, the contribution of electrostatic and hydrophobic forces to the process is an essential piece of the puzzle that needs to be elucidated^7^.

In this work, we have tackled these issues by attaching at the C terminus of gH625 a solubilizing host peptide proposed previously for other viral fusion peptides^27, 28^. This host-guest system greatly increases the solubility of the peptide and facilitates measurements of binding to lipid bilayer model membranes from aqueous solution. The presence of a flexible linker between the guest and host moieties does not alter the interactions with the membrane. Although the lysine tails are not physiological, they provide a useful tool to manipulate the aggregation state of the attached hydrophobic sequence.

Moreover, recently great attention has been dedicated to the key role of multivalency as an important parameter to be modulated for enhancing binding strength and hence for developing novel assemblies with potential biomedical applications^29–31^. Multivalency will result in an increased local concentration of the active peptide at the site where it is supposed to interact with the cellular components; thus enhancing its binding to targets. Moreover, multivalent interactions are likely to be much stronger than the corresponding monovalent interactions and are often involved in the regulation of physiological processes. We have examined this hypothesis through the study of gH625 dimers and monomers; this study provides valuable insights into the mechanism of uptake of nanosystems such as quantum dots, nanoparticles, liposomes where several to many peptides are covalently bound to their surface to achieve penetration. This point, in fact, plays a key role in view of the desire to construct more complex nanosystems which are likely to perform an increasingly significant task in research fields such as supramolecular, medicinal, and material chemistry.

In this paper we have used a combination of biophysical techniques to advance our understanding of the mechanism of interaction with membranes which will be useful for further exploiting gH625 as a delivery vector.

## Results

### Design of peptides

To analyze the interaction of the relatively hydrophobic peptide gH625 with membrane bilayers, we designed several peptides containing the host-guest system proposed by Han & Tamm^32^, which allow: (i) peptides to be soluble in aqueous buffers up to concentrations needed for fluorescence experiments; (ii) peptides to be monomeric in aqueous buffers to facilitate the analysis of binding equilibria; (iii) host and guest segments interactions to be minimal; (iv) to study the effect of multivalency (Table 1). The host peptide used in this study is named H7: GCGKKKK. The very polar nature of the host peptide guarantees the good solubility in aqueous buffer of the guest peptide and allows the peptide to remain monomeric in solutions of low ionic strength. The four consecutive lysines facilitate the binding of the guest peptide to lipid bilayers. The linker region consisting of the sequence GCG was incorporated into the host peptide. Being conformationally flexible, this segment fulfils our need to uncouple the secondary structure induction across the host and guest peptide boundary. Furthermore, the presence of a cysteine in the H7 sequence allowed the formation of a dimer and thus the study of multivalency, and at the same time warrants that both the N-terminus of the monomeric units remain free for membrane interactions as gH625 is known to enter the bilayer from its N-terminal side^6^.

**Table 1:**
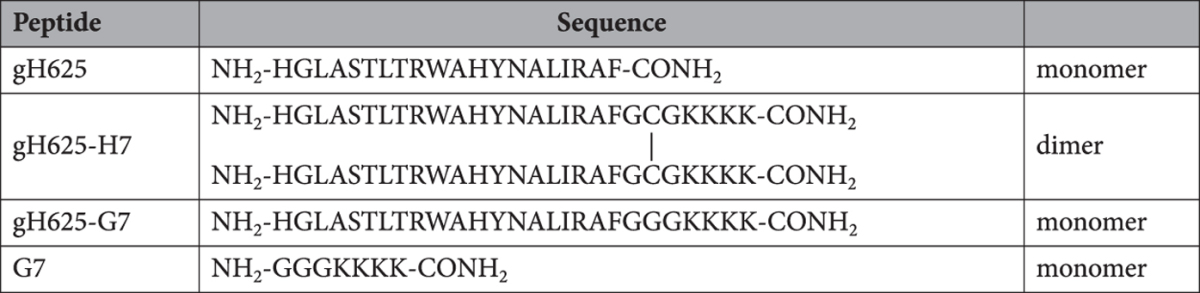
Peptide sequences.

### Lipid systems

In order to understand the interaction with membrane bilayers we prepared large unilamellar vesicles (LUVs), small unilamellar vesicles (SUVs) and giant unilamellar vesicles (GUVs) as reported in the experimental section and according to the experiment to perform. The vesicles components used in our experiments are: 1,2-Dioleoyl-sn-glycero-3-phosphocholine (DOPC), 1,2-dioleoyl-*sn*-glycero-3-phospho-(1′-*rac*-glycerol) (DOPG), Chloroform (Chol). The lipid compositions used are: DOPC/Chol 60/40 (mol/mol); DOPG/Chol 60/40 (mol/mol) and DOPG. The choice of the lipidic composition was meant to better dissect the effect of the charge on the interaction (DOPG was selected as negative lipid and DOPC as a zwitterionic lipid) and to study the role of the cholesterol; in particular, DOPG/Chol and DOPC/Chol were selected for analysing the effect of the peptides on liquid ordered phases, in comparison with the liquid disordered fluid domains (DOPG). Moreover, although DOPG is not usually found in eukaryotic cells, we selected this lipid also because we are interested in the application of gH625 for drug delivery with a particular focus on cancer therapeutics; it is in fact, widely known that cancer cells are characterized by a higher content of anionic lipids in the outer leaflet of the membrane^33^, and this could be envisaged as a valuable strategy for enhancing targeting. Cholesterol is usually found in eukaryotic cells and is responsible of the higher rigidity of membranes. The selected lipids are used at a temperature higher than their melting temperatures, thus they are expected in their fluid state; moreover, the addition of cholesterol reduces their fluidity making them stable as shown by the experiments on membrane fluidity reported below.

### Membrane fluidity

In order to characterize LUVs used in this study and to evidence modifications of membrane fluidity induced by gH625, gH625-H7 and gH625-G7, we used the fluorescent probe Laurdan^34, 35^. Laurdan inserts into membranes and distributes equally between lipid phases; its emission can shift from 440 nm in the ordered lipid phase to 490 nm in the disordered lipid phase; the parameter denoted Generalized Polarization (GP) is usually used to quantify the change^34^. The emission spectra of LUVs composed of DOPC/Chol 60/40 (mol/mol), DOPG/Chol 60/40 (mol/mol) and DOPG clearly indicate the presence of ordered phases at 25 °C; the spectra were reproducible after 24 h further supporting that the LUVs are stable and not leaky in our conditions. The data clearly confirm that DOPG LUVs are more fluid than LUVs containing cholesterol.

The analysis of the GP values (Table 2) clearly shows a higher value in presence of cholesterol (GP = 0.36 for DOPG/Chol and GP = 0.27 for DOPC/Chol), which is a consequence of formation of the liquid-ordered phase (Lo) rather than the disordered phase (Ld) which is the characteristic phase for the pure system (GP = 0.13 for DOPG). The reported experiments were performed also at 37 °C but results similar to 25 °C were obtained for cholesterol containing liposomes while DOPG LUVs present a spectrum shifted towards the red with a GP value of −0.061, indicative of a significant fluidity. No change was observed at 25 °C after 24 h (data not shown), which together with the dynamic light scattering experiments reported below, clearly indicate that our liposomes are stable.

**Table 2:** Membrane fluidity evaluation using the generalized polarization (GP) value calculate as GP = (I_440_−I_490_)/(I_440_+I_490_).

	No peptide 37 °C	No peptide 25 °C	gH625	gH625-H7	gH625-G7
DOPG/Chol 60/40 (mol/mol)	0.35	0.36	0.36	0.48	0.40
DOPG	−0.061	0.13	0.10	0.18	0.11
DOPC/Chol 60/40 (mol/mol)	0.27	0.27	0.28	0.34	0.29

The fluidity of the membrane was also probed in the presence of the three peptides at 25 °C. The presence of the peptides induced a change in the fluorescence emission intensity in all conditions. The GP parameter allowed to quantify the effect of the peptides (Table 2). In the case of gH625 the GP did not vary significantly while we observed changes for gH625-H7 and gH625-G7. The greatest changes were observed for gH625-H7 with the most significant change in DOPG/Chol where GP = 0.48. Thus, gH625-H7 showed an ordering effect on LUVs containing cholesterol which could be explained as an ability to induce the formation of ordered domains.

### Ability to induce lipid mixing and membrane leakage

**Figure 1:**
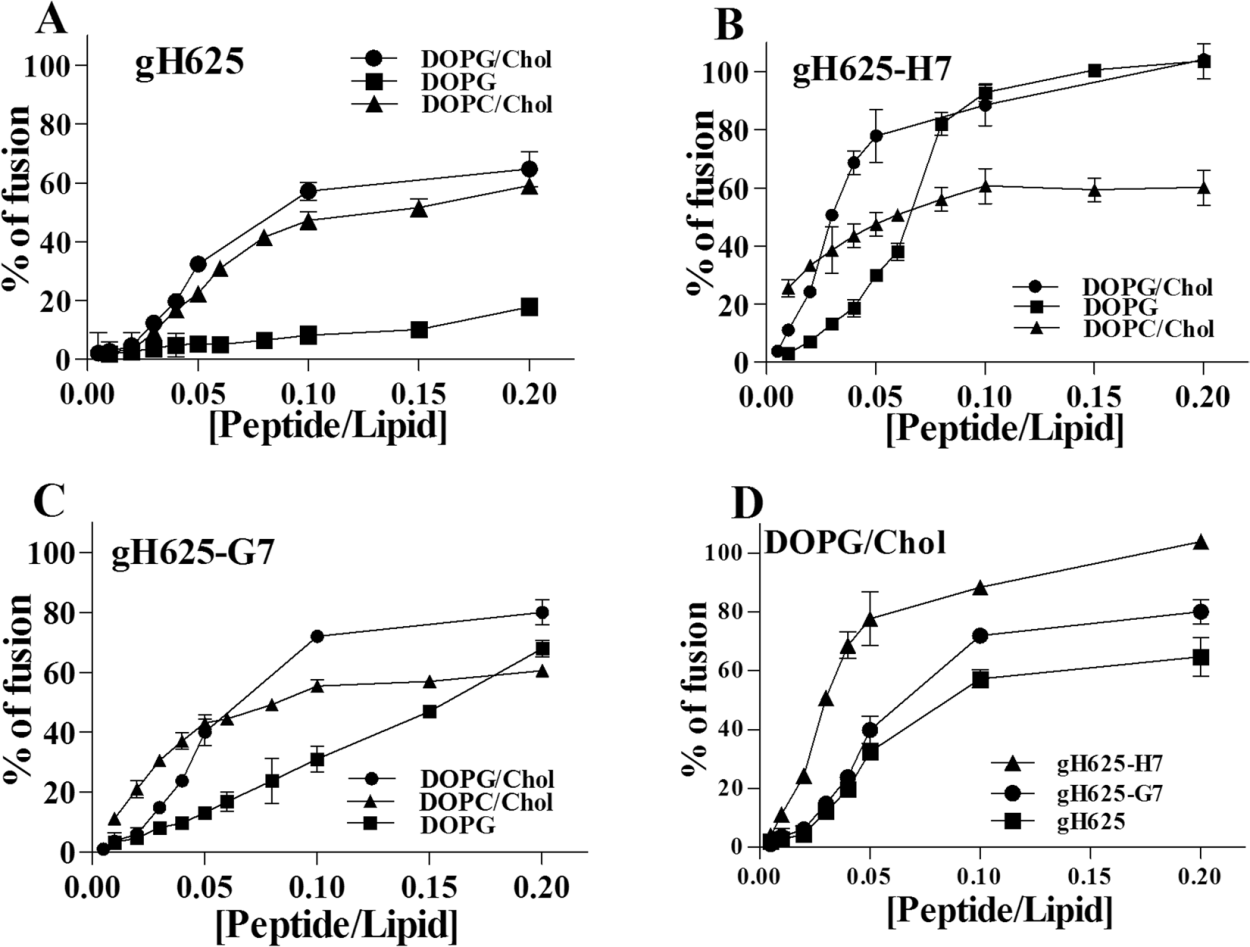
Peptide-promoted membrane fusion as determined by lipid mixing of gH625 (**A**), gH625-H7 (**B**) and gH625-G7 (**C**) in LUVs composed of: DOPG, DOPG/Chol 60/40 (mol/mol) and DOPC/Chol 60/40 (mol/mol). Two populations of LUVs, one labelled with 0.6 mol% of NBD (donor) and Rho (acceptor) and one unlabelled were mixed and the variation in NBD fluorescence due to membrane fusion was determined. Lipid concentration is 0.1 mM. Percentage of membrane fusion is reported as a function of the peptide/lipid ratio and each trace represents an average of three independent experiments. Panel D reports the peptide-promoted membrane fusion of the three peptides in DOPG/Chol LUVs 60/40 (mol/mol).

In order to gain insight into the ability to interact and perturb the lipid membrane, nitrobenzoxadiazole (NBD) and Rhodamine (Rho) were used as the donor and acceptor of fluorescence energy transfer. Dilution of labelled vesicles caused by peptide induced membrane fusion, determines a larger average distance between fluorescent and quenching lipids and thus a reduction in the fluorescence energy transfer efficiency. Figure 1 (panels **A,B,C**) shows the results of lipid mixing assays for the 3 peptides in LUVs of different compositions (DOPG/Chol, DOPG and DOPC/Chol). As expected the peptide gH625 is less active than the other two in DOPG because this is a negatively charged lipid and gH625 is strongly hydrophobic thus preferring zwitterionic lipids. On the contrary, the interaction of both gH625-H7 and gH625-G7 with DOPG is significantly stronger as a result of electrostatic interactions. The three peptides interact more strongly with LUVs containing cholesterol, confirming the preference for more rigid membranes. In Fig. 1D we clearly observe the significantly higher activity of gH625-H7 in DOPG/Chol LUVs compared to the other peptides.

**Figure 2:**
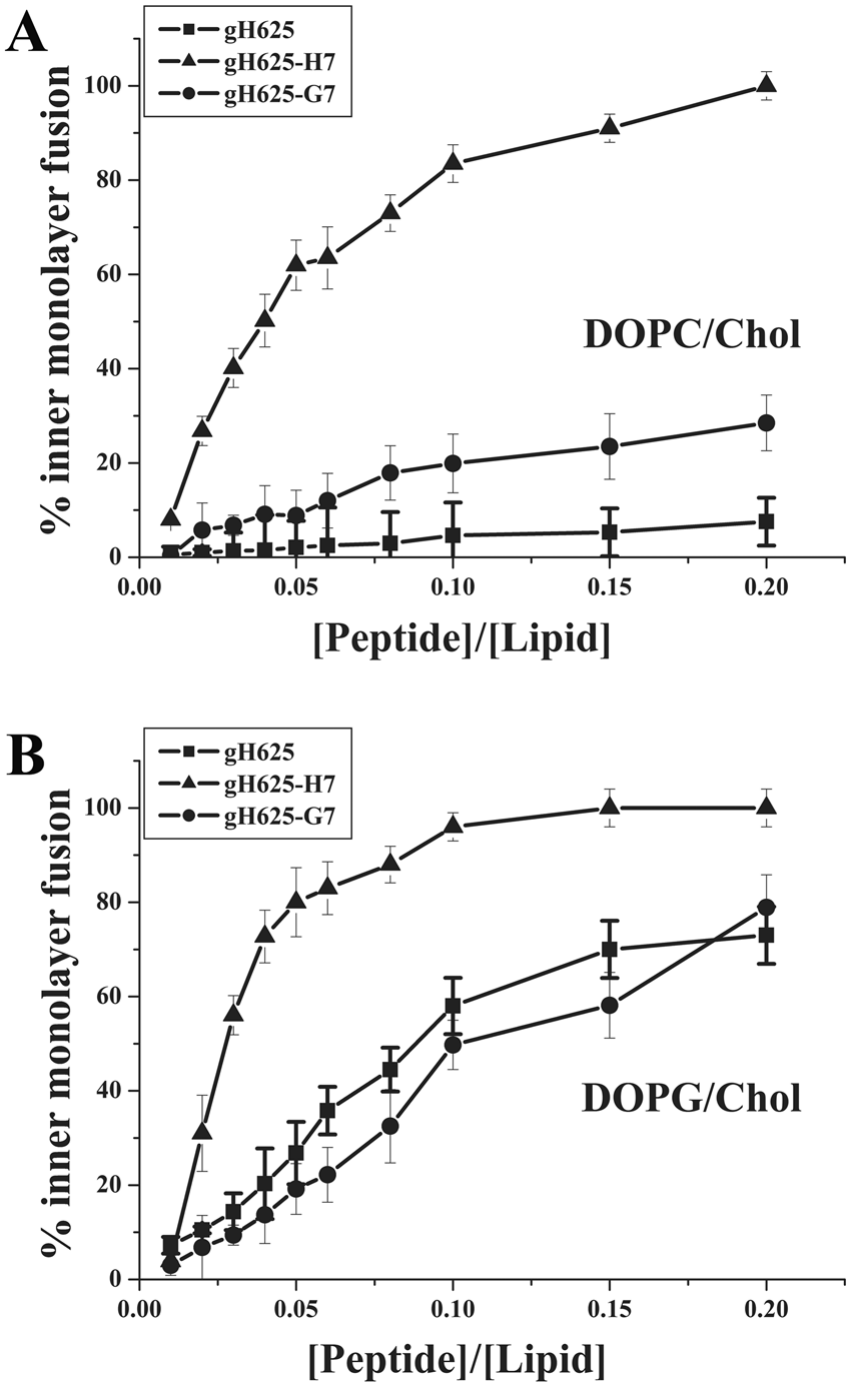
Inner monolayer in DOPC/Chol 60/40 (mol/mol) (**A**) and DOPG/Chol 60/40 (mol/mol) (**B**). Two populations of LUVs, one labelled with 0.6 mol% of NBD (donor) and Rho (acceptor) and one unlabelled were mixed; the NBD located on the outer monolayer of LUVs, was completely reduced by treatment with sodium dithionite (100 mM) and the variation in NBD fluorescence due to membrane fusion of inner monolayer was determined. Lipid concentration is 0.1 mM. Percentage of inner monolayer fusion as a function of the peptide/lipid ratio is reported and each trace represents an average of three independent experiments.

The extent of lipid mixing between only the inner monolayers of vesicles in solution, labelled with NBD and Rho, was determined adding an aqueous reducing agent (dithionite) which eliminated the NBD fluorescence from the vesicle membranes’ outer monolayer. Figure 2A shows that gH625 and gH625-G7 are inducing only a small fusion of the inner monolayer of DOPC/Chol LUVs which is compatible with hemi-fusion, while gH625-H7 produces a complete fusion also of the inner monolayer. Figure 2B shows that in DOPG/Chol also the other two peptides are able to induce a significant inner monolayer fusion; we observe complete fusion of the inner monolayer for gH625-H7.

Pore formation is not strictly correlated to vesicle fusion events; thus, leakage experiments which monitor the mixing of internal vesicle components are widely employed. In particular, vesicles co-encapsulating 8-aminonapthalene-1,3,6 trisulfonic acid (ANTS) and p-xylene-bis-pyridinium bromide (DPX) were titrated with peptides and release of ANTS and DPX as a result of vesicle exposure to peptides, was used as a measure of the membrane-disruptive capacity of the peptides through the formation of transient pores^36^; an increase in fluorescence intensity of ANTS should be observed when pore formation takes place after peptide addition because quenching by DPX will be diminished. No content-mixing occurs (data not shown) over the same P/L range where substantial outer and/or monolayer lipid-mixing occurs within our systems.

These data support the view that gH625 and its modifications are able to deeply penetrate inside the bilayer without any associated leakage event, which may be relevant for the direct penetration mechanism of cell entry, or for the steps of endosomal membrane translocation or endosomal escape. Vesicle fusion events not accompanied by leakage of the aqueous contents of the vesicle have also been reported by others for cell penetrating peptides^37^. Another important issue to keep in mind is that often membrane leakage is correlated to cellular toxicity as for antibacterial peptides, thus the absence of leakage is key for selecting sequences useful for applications in drug delivery. The presence of the lysine residues is not sufficient to induce any leakage as it happens in antimicrobial peptides (AMPs)^38^.

### Tryptophan fluorescence emission analysis

**Figure 3:**
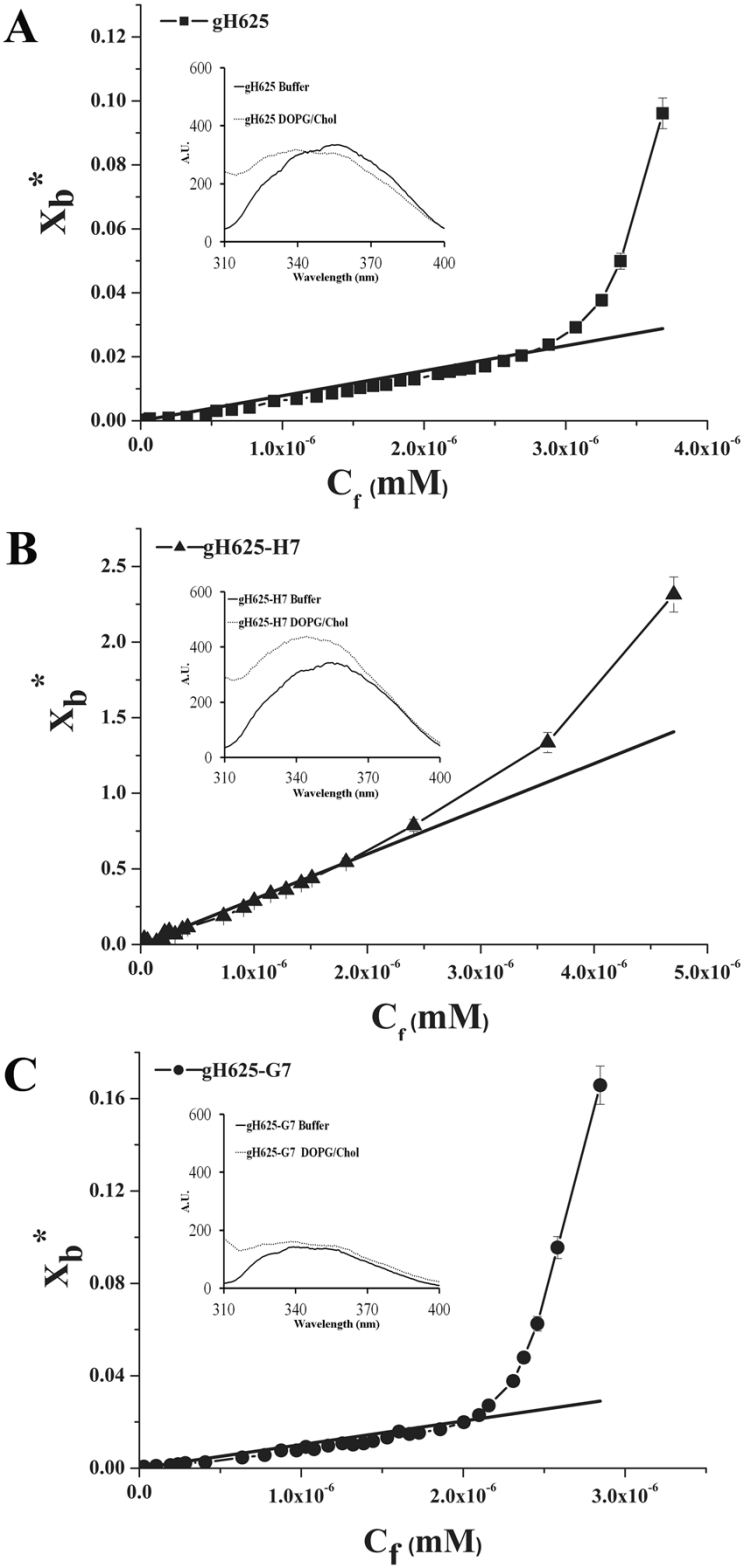
Binding isotherms in DOPG/Chol 60/40 (mol/mol) obtained plotting X_b_^*^ versus C_f_ for gH625 (**A**) and gH625-H7 (**B**) and gH625-G7 (**C**). In the inserts are reported the fluorescence spectra of the tryptophan in buffer and in DOPG/Chol 60/40 for each peptide; the peptide concentration for the spectra is 4 μM and the lipid/peptide ratio is 200.

The intrinsical fluorescence due to the tryptophan environmentally sensitive residue present in the middle of the sequence^39^, allowed to evaluate their degree of penetration into the membrane bilayer. We compared the fluorescence emission spectra in the presence of DOPG/Chol SUVs with that in buffer (Figure 3) and the observed changes in the spectral properties, suggested the location of the tryptophan residues of each of them in a less polar environment upon interaction with lipids. Emission intensity was enhanced and the maxima shifted to lower wavelengths; the observed blue shifts (344 to 336 nm) are typical of amphiphilic tryptophan-containing peptides which can also oligomerize inside the membrane^39^. We observed a small shift, which likely point toward a change in local environment upon replacement of tryptophan/polypeptide contacts with tryptophan/lipid contacts. The results obtained support the location of the tryptophan inside the membrane and the presence of oligomerization processes inside the bilayer^6^.

Furthermore, the increased fluorescence of tryptophan binding to membrane phospholipids was exploited to obtain the binding isotherms for the three peptides and to calculate the partition coefficients. The concentrations of peptides used were low enough to produce negligible aggregation in the aqueous phase and were presumed to not disrupt the bilayer structure. Figure 3 reports the plot of the moles of bound peptide per moles of lipid as a function of the free peptide concentration, according to the following equation:

1 $${X}_{b}^{\ast }={{\rm{K}}}_{{\rm{p}}}{{\rm{C}}}_{{\rm{f}}}$$

where K_p_ is the apparent partition coefficient in units of M^−1^, X_b_^*^ is the molar ratio of bound peptide per total lipid (considering that the peptides were initially partitioned only over the outer leaflet of the SUV)^40^ and C_f_ is the equilibrium concentration of the free peptide in solution^6^. The fluorescence intensities of the free (F_0_) and bound (F_∞_) peptides, were used to calculate the fraction of membrane-bound peptide, f_b_, from the formula

2 $${{\rm{f}}}_{{\rm{b}}}=({\rm{F}}-{{\rm{F}}}_{0})/({{\rm{F}}}_{\infty }-{{\rm{F}}}_{0})$$

where F represents the fluorescence of the peptide at a given added lipid concentration. The equilibrium concentration of free peptide in solution, C_f_, and the extent of peptide binding X_b_ were obtained from f_b_^6, 41, 42^.

A non-linear relationship typical of peptides that self-associate at membrane surfaces upon partitioning was observed^42^. The obtained shape is characterized by a pronounced flattening at the origin followed by a steep rise, which was considered indicative of a process whereby peptides first incorporate into the membrane and then aggregate there within; in this situation, the total extent of incorporation (X_b_^*^) slowly increases until a critical concentration is reached, where substantial internal aggregation starts to develop^40, 42^. This is further supported by the fact that there is no aggregation in water in the experimental conditions used for this assay. The shape of the graph obtained for gH625-H7 clearly indicated a stronger interaction.

The surface partition coefficients K_p_ were extrapolated from the initial slopes of the curves to C_f_ values of zero;^42^ curves are shown in Figure 3 and K_p_ values are reported in Table 3. The K_p_ value obtained for gH625 in DOPG/Chol is 7.8 10^3^, indicating that the tryptophan in gH625 is embedded in or adsorbed on the bilayer; this value is lower compared to the one previously obtained for gH625 in PC/Chol liposomes^6^. We expected this difference since DOPG/Chol liposomes are negatively charged compared to PC/Chol that are zwitterionic. The K_p_ value for gH625-G7 and gH625-H7 is higher and respectively is 10.2 10^3^ and 290 10^3^ indicating that the tryptophan residue of these two peptides is more strongly interacting with the bilayer.

**Table 3:** Partition coefficients (Kp) and Stern-Volmer (Ksv) quenching constants.

	gH625	gH625-H7	gH625-G7
K_p_ in DOPG/Chol	(7.8 ± 0.2) 10^3^	(290 ± 5) 10^3^	(10.2 ± 0.5) 10^3^
K_sv_ (M^−1^) in buffer	10.81 ± 0.18	13.32 ± 0.38	12.21 ± 0.16
K_sv_ (M^−1^) in DOPG/Chol	8.07 ± 0.38	3.66 ± 0.09	4.74 ± 0.12

**Figure 4:**
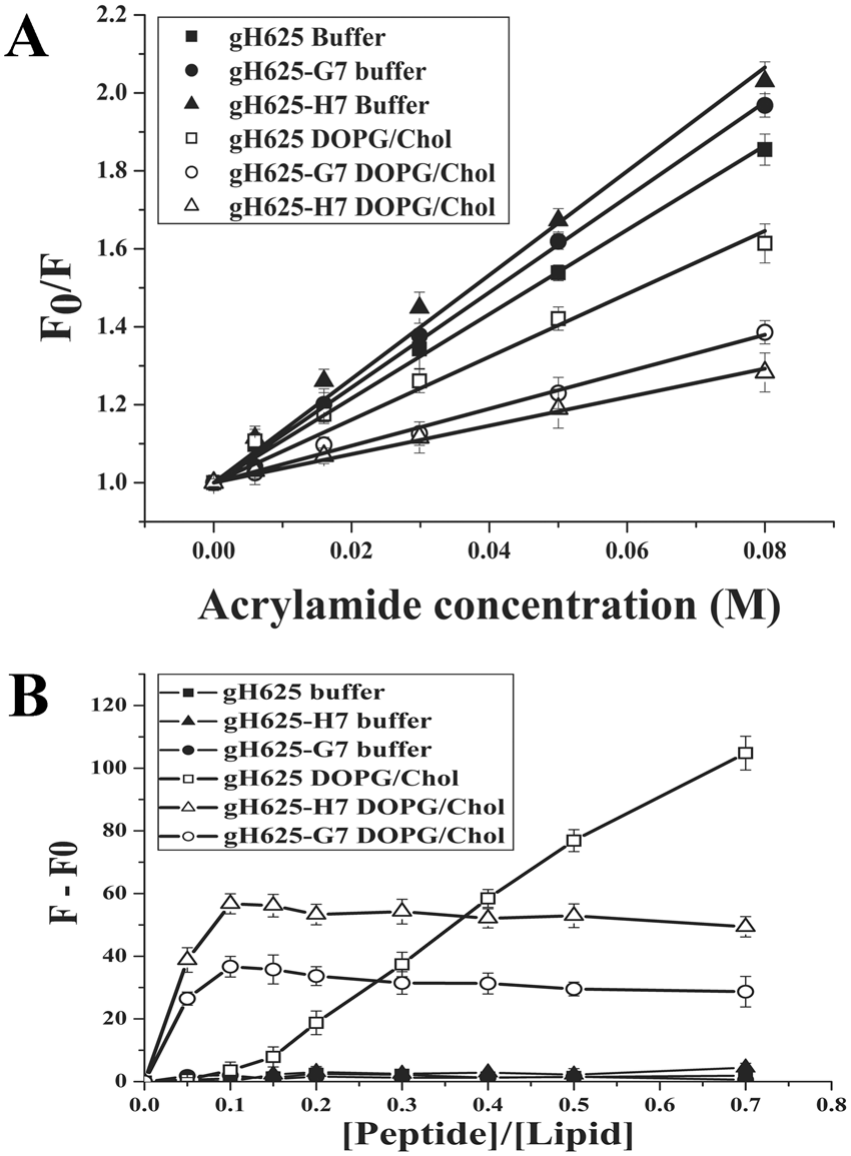
Panel A reports the quenching of tryptophan by acrylamide. Stern-Volmer plots of acrylamide quenching of gH625, gH625-H7, gH625-G7 in buffer (**closed symbols**) and in LUVs composed of DOPG/Chol 60/40 (mol/mol) (**open symbols**). Peptide concentration is 4 μM. Panel **B** reports the quenching of tryptophan by ThT as a function of the peptide/lipid ratio of gH625, gH625-H7, gH625-G7 in buffer (**closed symbols**) and in 0.1 mM LUVs composed of DOPG/Chol 60/40 (mol/mol) (**open symbols**).

The observed changes in the tryptophan emission upon binding of peptides to lipid vesicles indicate their insertion into the hydrophobic region of the bilayers, with the peptide gH625-H7 showing the greatest modifications. Thus, we analysed the accessibility of the tryptophan residues of membrane-bound peptides towards acrylamide, a neutral, water-soluble quencher, which is unable to penetrate into the hydrophobic core of the lipid bilayer; thus, tryptophan residues buried inside the membrane are less strongly quenched by acrylamide. Figure 4A shows the Stern-Volmer plots for the quenching of tryptophan by acrylamide, in the absence and presence of LUVs. In the presence of LUVs, we observe less decrement in fluorescence intensity which was indicative of a less accessible tryptophan. The values for K_sv_ were lower for gH625-H7 (Table 3) in LUVs, suggesting that tryptophan was more buried in the bilayers, becoming more inaccessible for quenching by acrylamide. Comparison of the plots (Figure 4A) and of the values of Ksv (Table 3) confirms that the tryptophan in gH625-H7 is more deeply inserted compared to the native sequence (gH625).

### Peptide aggregation

The peptide aggregation state was determined using the Thioflavin T (ThT) experiment^43, 44^, results are shown in Figure 4B. The three peptides are not aggregated in buffer as evidenced by the fact that the fluorescence remained unaltered after the addition of the peptides (see closed symbols). On the contrary, they all caused a progressive aggregation in LUVs as the fluorescence change increased dramatically. In particular, a drastic increase of fluorescence is obtained for gH625-H7 and gH625-G7 already at low peptide/lipid ratios, indicating that the two peptides oligomerize significantly in liposomes. The results obtained for gH625 indicate a lower aggregation at low peptide/lipid ratio and a significant increase at higher ratios with an increased fluorescence.

### Dynamic light scattering (DLS)

**Figure 5:**
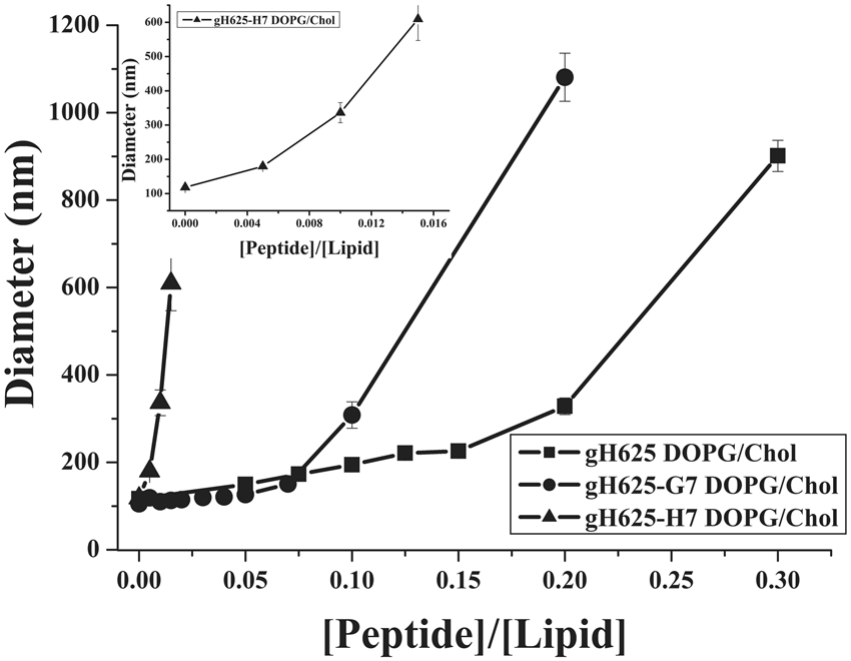
Dynamic light scattering of LUVs composed of DOPG/Chol 60/40 (mol/mol) after the addition of gH625, gH625-H7 and gH625-G7. Lipid concentration is 0.1 mM. Diameter (nm) is reported as a function of the peptide/lipid ratio. The insert reports an enlargement of results obtained for gH625-H7.

DLS was employed to examine whether the peptide affects the dimensions of LUVs (Figure 5). Hydrodynamic diameter was calculated before and after the addition of the three peptides. The mean diameter is 100 nm for LUVs alone, while it increases with addition of peptides. The greatest increase was observed for gH625-H7 and already at a peptide/lipid (P/L) ratio of 0.004 (mean diameter 200 nm) (Figure 5, insert). Indicating that a lower quantity of peptide is necessary for fusion of vesicles by gH625-H7. As expected the PDI increases at higher P/L ratio indicating a non-homogenous sample when aggregated. Mean diameters and polydispersity indexes (PDI) for each measure are reported in Supplementary material.

### Binding affinity of the peptides to lipid bilayers measured by Surface Plasmon Resonance (SPR)

**Figure 6:**
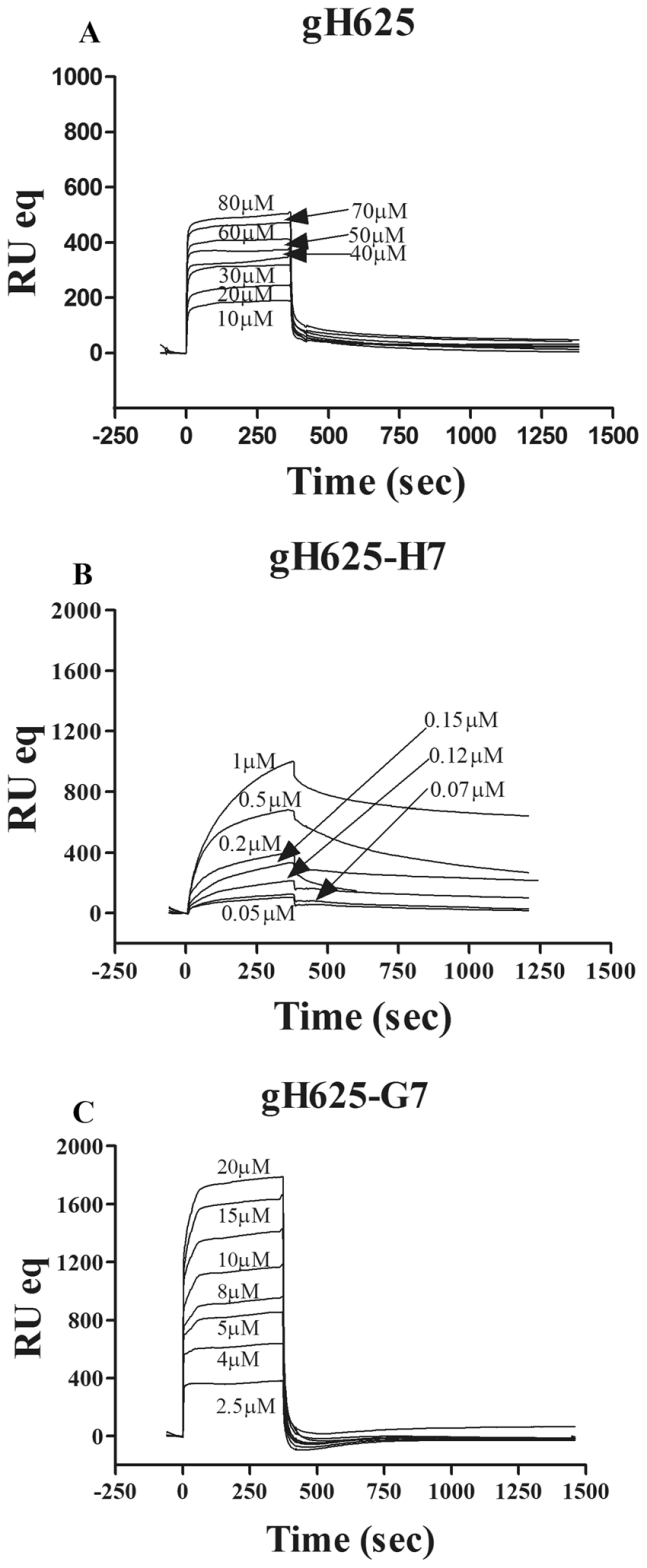
Sensorgrams of the binding between various concentrations of gH625 (**A**), gH625-H7 (**B**), and gH625-G7 (**C**) with liposomes formed of DOPG/Chol 60/40 (mol/mol). The sensorgrams were obtained with a L1 chip which contains hydrophobic alkanethiol chains to bind liposomes.

To investigate the mode of action of gH625-H7 and gH625-G7 and for comparisons with gH625^6^, we used SPR. In a previous paper^7^ we also reported the data for two control peptides: the melittin and the HIV fusion peptide (gp41-FP). Figure 6 reports the obtained sensorgrams of the binding and an increase of the RU signal intensity as a function of the peptide concentration can be observed. The analysis of the shape reveals different binding kinetics among the three peptides. In particular, all the sensorgrams obtained for gH625^7^ and gH625-G7 indicate the binding to the lipid surfaces is characterized by an initial fast association process which then slows down considerably towards the end of the peptide injection. The dissociation follows a similar trend, characterized by the signal falling rapidly at the end of injection when the peptide is no longer present and the buffer flow removes free or weekly bound peptide. The sensorgrams obtained for gH625-H7 evidenced significant differences both in the initial association and in the dissociation processes as can be seen from Figure 6B. The fact that the sensorgrams did not return to zero, indicates that the peptide remained appreciably bound to the surface or inserted into the hybrid bilayer membrane.

It was not possible to obtain a good fit (X^2^ > 100) with the 1:1 Langmuir binding model which supported the hypothesis that this model does not represent the lipid binding mechanism of membranotropic peptides^7, 45, 46^. On the contrary, a significantly improved fit was obtained with the two-state reaction model^7, 45, 46^; the first step corresponds to the binding of the peptide to the surface, which may be driven by electrostatic forces; and the second to the insertion of the peptide into the hydrophobic core of the membrane. The average values for the rate constants obtained with the two-state model analysis are reported in Table 4 along with the affinity constant values (*K*_*A*_). The data clearly indicate the main influence on the overall binding constant of the fast association rate and slow dissociation rate of the first step. If this step corresponds to the electrostatic interaction, these results clearly indicate that electrostatic forces play an important role in the binding of membrane-active peptides especially for the presence of lysine residues. In this respect, it is interesting to note that gH625-H7 has a significantly higher K_1_ compared to the other peptides, indicative that the extra lysine residues responsible of the first step electrostatic interaction are key.

**Table 4:** Association and dissociation rate constants obtained for the L1 chip using the two-state model.

Peptide	k_a1_(1/Ms)	k_d1_(1/s)	K_1_(1/M)	k_a2_(1/s)	k_d2_(1/s)	K_2_	K_A_ (1/M)
gH625	(5.85 ± 0.01) 10^3^	(8.72 ± 0.08) 10^−2^	6.70 10^4^	(1.07 ± 0.01) 10^−3^	(2.61 ± 0.01) 10^−3^	0.41	9.45 10^4^
gH625-H7	(1.63 ± 0.02) 10^4^	(1.20 ± 0.04) 10^−2^	1.36 10^6^	(8.32 ± 0.03) 10^−3^	(2.14 ± 0.07) 10^−3^	3.89	6.63 10^6^
gH625-G7	(2.62 ± 0.07) 10^2^	(2.49 ± 0.02) 10^−2^	1.05 10^4^	(5.72 ± 0.04) 10^−7^	(1.00 ± 0.07) 10^−7^	5.72	7.09 10^4^

The affinity constants *K*_1_ and *K*_2_ are for the first (*K*_1_ = *k*_a__1_/*k*_d__1_) and for the second (*K*_2_ = *k*_a__2_/*k*_d__2_) steps respectively, and the affinity constant (*K*_A_), determined as (*k*_a__1_/*k*_d__1_) × (*k*_a__2_/*k*_d__2_), is for the complete binding process. S.D. are reported in parentheses.

### Secondary structure

**Figure 7:**
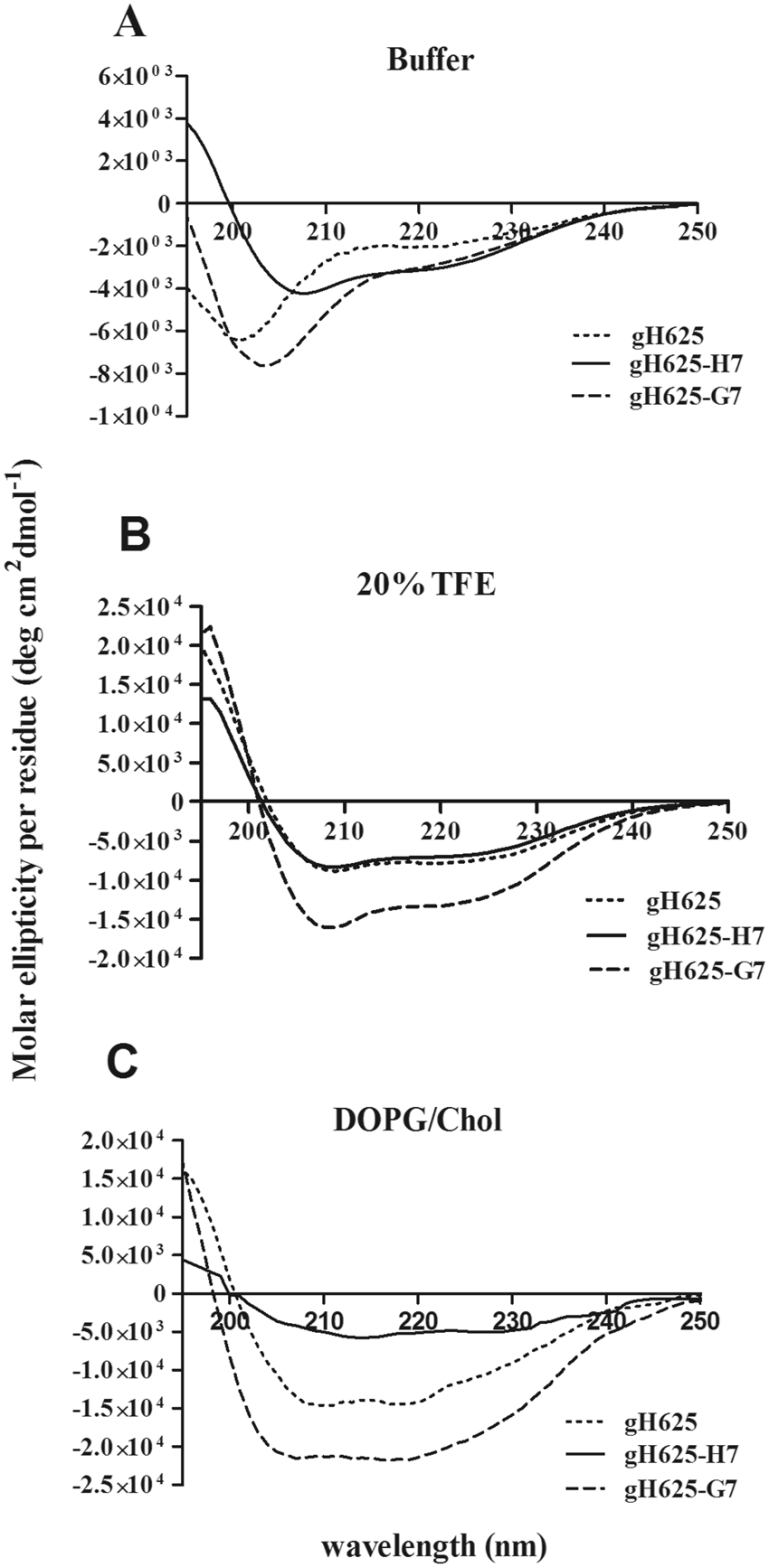
Circular dichroism spectra of gH625, gH625-H7 and gH625-G7 in buffer (**A**), 20% TFE (**B**) and DOPG/Chol 60/40 (mol/mol) LUVs (**C**). Peptide concentration used is 8 μM in all conditions tested and the peptide/lipid ratio is 0.05 in condition **C**.

The circular dichroism (CD) analysis was used to reveal changes of the secondary structure with respect to the parent peptide in different experimental conditions (Figure 7). As previously reported^7^, gH625 adopts a random coil structure in aqueous media, but forms a helix in membrane-mimetic environments^6, 9^. gH625 and gH625-G7 both exhibit a mainly random coil structure in buffer (Figure 7A); the calculations of helix content according to Chakrabartty *et al*.^47^ gives a 5% for gH625 and 7% for gH625-G7 with a transition to a α-helical structure with two negative bands at about 208 and 222 nm, in the helix inducing environment of trifluoroethanol (TFE); Figure 7B reports the spectra at 20% of TFE and the helix content are 22% for gH625 and 36% for gH625-G7. The dimer peptide (gH625-H7) shows a typical α-helical spectrum also in buffer (8% of helix) and in presence of TFE the percentage of helix is approximately 20%. The helix content is probably underscored because oligomerization processes are taking place in this condition. The CD spectra were also obtained in presence of DOPG/Chol. The spectra show that the three peptides are able to adopt an helical structure in lipidic environment with calculated percentages of helix corresponding to 37% for gH625 and 55% for gH625-G7; the spectrum of gH625-H7 in the same conditions was clearly indicating that oligomerization processes were taking place.

The ratio of the ellipticities at 222 and 208 nm can be utilized to distinguish between monomeric and oligomeric states of helices^48, 49^; when the ratio θ_222_/θ_208_ is lower than 0.8, the peptide is essentially in its monomeric state, and when the ratio exceeds the value of 1.0, it is in its oligomeric state. The data reveal that in presence of TFE and of lipids, gH625 adopts an α-helical conformation with the monomer/oligomer equilibrium shifted toward the oligomeric state with a ratio θ_222_/θ_208_ of approximately 0.9 in both conditions, while in buffer the ratio decreases to 0.5 indicating a monomeric state. The peptide gH625-G7 revealed a characteristic α-helix spectrum in TFE and lipids; the values of θ_222_/θ_208_ is 0.7 in TFE and 1.0 in lipids, indicating that the peptide is clearly in its oligomeric state in lipids. (Figure 7B,C). We repeated the experiment after centrifugation to remove eventual precipitated peptides from solution, however, the aggregates were molecular in size, and centrifugation had no effect on the spectra. The same analysis were performed on gH625-H7. The peptide adopts an α-helical conformation also in buffer, with a ratio θ_222_/θ_208_ of approximately 0.7, 0.9 and 1.2 in buffer, TFE and lipids. These data clearly confirm that also in TFE 20% oligomerization processes are taking place. The spectrum in lipids shows a sudden fall in molecular ellipticity which was also previously observed for other aggregating peptides^49, 50^. In conclusion, gH625-G7 is less oligomerized in TFE and this is probably correlated to the presence of the positive charges which prevent the electrostatic interactions between the peptides; while in membrane environment the positive charges are balanced by the electrostatic interactions with the head groups.

### GUVs image analysis and evaluation GUVs area

**Figure 8:**
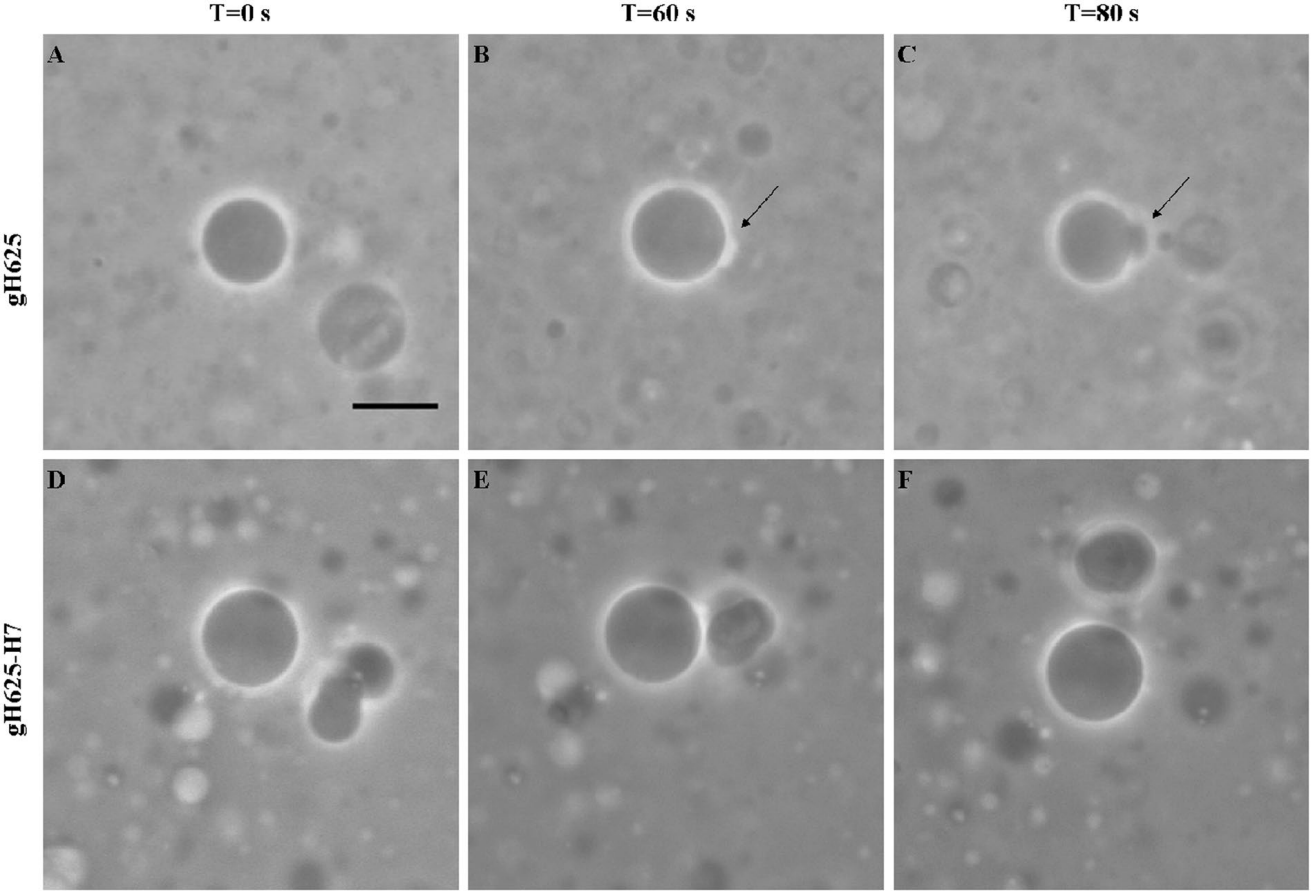
Representative differential interference contrast images of GUVs before and after addition of 5 μL of gH625 (**A**,**B**,**C**) or 5 μL of gH625-H7 **(D**,**E**,**F**). In all images out of focus GUVs are represented. Initial protrusion formation after 60 seconds from gH625 administration (**B**, **arrow**); late protrusion formation after 80 seconds from initial protrusion formation (**C**, **arrow**). Early fusion event as soon as gH625-H7 was added. Scale bar corresponds to 10 μm. The necessary quantity of gH625 or gH625-H7 was added to obtain a peptide/GUVs ratio of 0.5 under constant acquisition.

**Figure 9:**
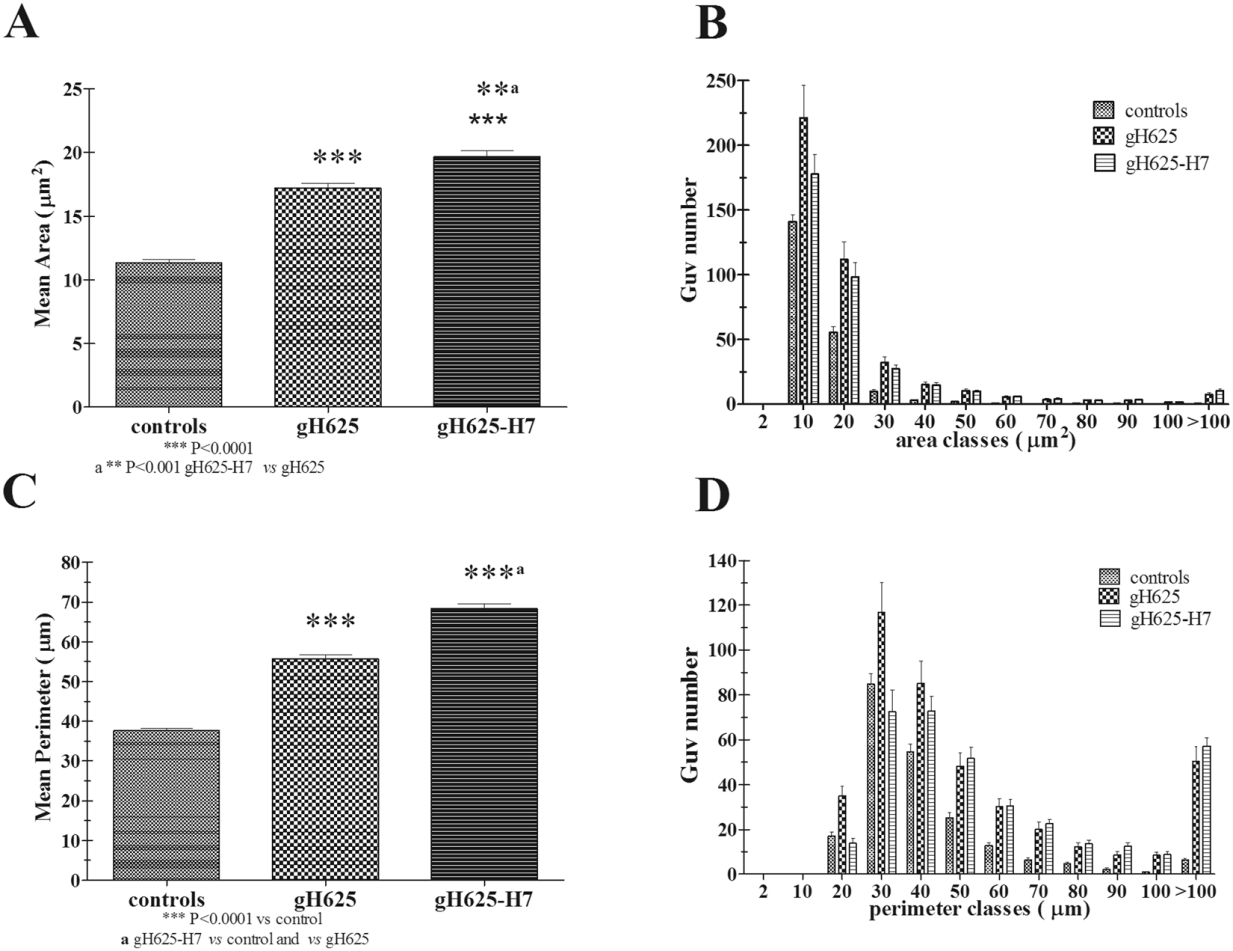
Mean area and perimeter of GUVs before (controls) and after treatment with gH625 and gH625-H7 (**A**,**C**); distribution of area and perimeter classes of GUVs before (controls) and after treatment with gH625 and gH625-H7 (**B**,**D**). Areas below 10 μm^2^ and perimeter below 20 μm were not considered since values may not be significant for very small vesicles.

GUVs appear dark against the background as a consequence of the difference in refractive index of GUVs contents (sucrose solution) and the surrounding medium (glucose solution). A white halo appears around the vesicle. Formation of pores and of protuberances may be thus evidenced. GUVs appear under differential interference contrast (DIC) round shaped with an average smaller area compared to GUVs treated with gH625 (Table 5, Figure 8). The addition of gH625 determines the protrusion of blebs (almost one per GUV) which were observed to be stable in time (Figure 8). Vesicles retained their halo effect during observation time, indicating that in these experimental conditions peptides do not cause any perforation of the membranes that would allow exchange of outer and inner solutes and thus reduction of the halo intensity. The addition of gH625-H7 to GUVs produced a similar effect of gH625 with the only difference that it induces an enhancement of the GUVs fusion tendency (Figure 8). gH625 and gH625-H7 enhance the mean area and perimeter of GUVs (Figure 9A,C). The area and perimeter distributions of GUVs (Figure 9B,D), respectively suggest that gH625 or gH625-H7 administration strongly increases the frequency of larger GUVs, indicating that peptides enhance GUVs’ fusion. Main shape descriptor analysis confirms that the addition of gH625 or gH625-H7 induces an increase in area and perimeter also indicating a decrease in circularity and roundness parameters (Table 5).

**Table 5:** Mean shape descriptors before and after the gH625 treatment. Circularity = 4π × [Area]/[Perimeter]^2^ with a value of 1.0 indicating a perfect circle. As the value approaches 0.0, it indicates an increasingly elongated shape. Roundness = 4 × [Area]/π × [Major axis]^2^.

Mean	GUVs	GUVs+gH625	GUVs+gH625−H7	Var % gH625	Var % gH625−H7
Circularity	0.12	0.11	0.08	−8.33	−33.33
Roundness	0.66	0.59	0.54	−10.60	−18.18

Var % = percentage of variation of gH625 or gH625-H7 versus GUVs alone as is for each parameter % = [(treated GUVs − control GUVs)/control GUVs]*100.

### Acceptor photobleaching fluorescence

**Figure 10:**
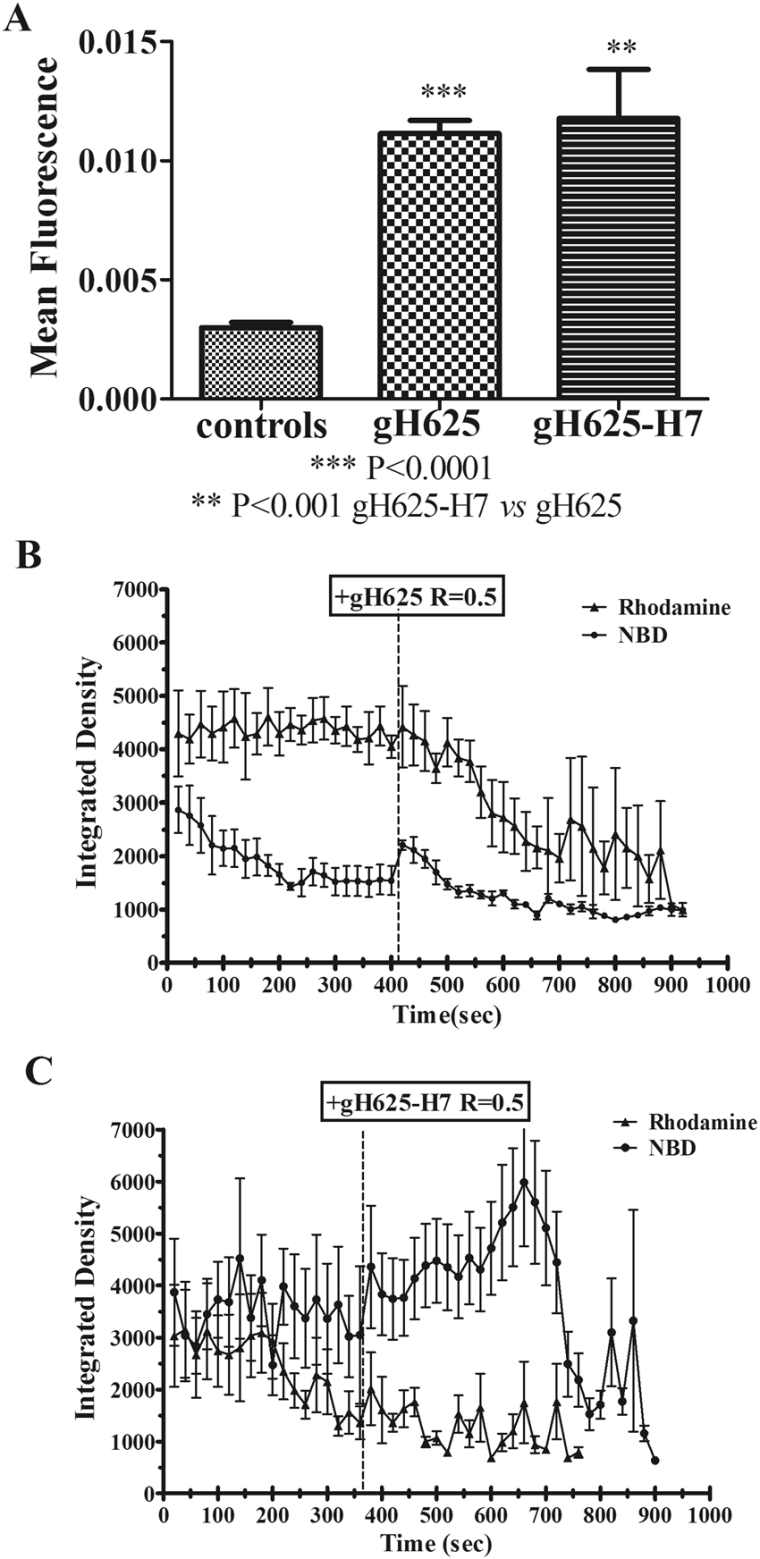
Mean fluorescence of GUVs suspension resulting after gH625 or gH625-H7 administration, calculated by the AccPbFRET plug in of ImageJ. The FRET efficiency *E* is 0.729, calculated as follows: *E* = 1 − *(F Donor quenched by acceptor/F Donor de quenched)* (**A**). Time course of FRET effect due to gH625 or gH625-H7 administration to GUVs. Values are expressed as integrated density, defined as the mean gray value per the mean area. Each time point represents the same field acquired in both channels. Each data set represents the mean of at least 10 GUVs for each field (**B**,**C**).

The administration of gH625 causes GUVs’ fusion, hence increasing distance between the donor (NBD) and acceptor (Rhodamine) fluorophores, determining the dequenching of the donor, which results in a 3.71-fold increase of its fluorescence compared to GUVs alone (Figure 10A). The administration of gH625-H7 induces an increase in fluorescence of 3.92-fold compared to the fluorescence of GUVs alone (Figure 10A). The time course for both peptides shows the FRET effect on GUVs due to peptide administration (Figure 10B,C).

## Discussion

The mechanism used by CPPs to cross the membrane is a controversial matter of debate although it is well established that the main internalization mechanism is endocytosis. gH625 is particularly interesting for its ability to translocate cargo macromolecules without involving essentially the endocytic route. A preferential internalization mechanism by an endocytosis independent mechanism was in fact, demonstrated *in vitro* ^5, 10, 15^.

The fact that gH625 has to cross the cell membrane in order to deliver intracellularly a cargo, makes necessary investigating its interactions with lipids to study its mechanism of action and eventually side effects. Liposomes are useful model systems to study membrane interactions; moreover, not possessing the endocytic machinery, they represent a good model to test direct translocation through the membrane avoiding to perform cell uptake studies at 4 °C (for inhibition of endocytic pathways) when the lowering of the temperature decreases membrane fluidity and thus also affects direct translocation processes. In addition, lipid composition of eukaryotic cell membranes is very complex and dynamic, while liposomes allow a simplification enabling to monitor the initial steps of cell entry, binding to membrane and translocation through it as well as to simultaneously assess the organization and dynamics of the membrane and the relationship between the peptide structure and ability to translocate.

Penetration of CPPs into membranes is a dynamical process and has different stages and pathways. One of the key issues is the penetration mode, and whether penetration occurs due to a collective effect of several peptides or only one. Thus, it is necessary to characterize the binding mode of a single peptide and of dimers to the bilayer and to evaluate the contribution of multivalency and hydrophobicity on the ability to interact with lipid membranes. In this study, we compared the effects of the native sequence with those of modified ones gH625-H7 (dimer) and gH625-G7 (monomer), respectively.

The initial interaction of a carrier peptide with the membrane is a prerequisite for the subsequent membrane translocation process. gH625 translocates and can work as a vector to introduce proteins or other cargo molecules inside cells. The results obtained from this work show an ability of gH625 monomers and dimers to interact strongly with membrane bilayers with a preference for zwitterionic or negatively charged lipids correlated to their charge. In fact, gH625 interacts more strongly with zwitterionic membranes while both gH625-H7 and gH625-G7, bearing the positive charges at the C-terminus, have a strong preference for negatively charged lipids. The fact that the positive charges are located at the C-terminus is not affecting their interaction with the bilayer which was previously shown to directly involve the N-terminus of the peptide^6^. Our results are clearly in agreement with the fact that membrane interactions depend on the balance between hydrophobicity and charge; in fact, hydrophobic domains are essential for interactions and together with electrostatic contacts are believed to play a key role to promote cellular uptake.

The strong interaction with the lipid bilayer causes a local perturbation and enables it to cross the membrane by a physical mediated mechanism not involving pore formation^5^. This is clearly evident from the fusion, inner monolayer fusion and leakage experiments; in fact, although the three peptides have a different interaction with bilayers, with gH625-H7 favouring also the fusion of the inner monolayer, the analysis of leakage experiments shows that none of them is inducing pore formation. This energy-independent translocation mechanism is initiated by peptide interactions with the membrane lipids, which lead to a perturbation of the membrane without pores; this of course does not exclude a possible internalization by an endocytic route in some conditions. On the contrary, for most CPPs endocytosis is the main mechanism of uptake and usually these leaking-inducing CPPs also present more toxicity to mammalian cells.

Differences between gH625 and other CPPs can be related to the affinity for membrane lipids. Peptides with higher affinity have a greater propensity to be internalized by a non-endocytic mechanism, while lower affinities can favor endocytic uptake. Because of the strong correlation between gH625-lipid membrane affinity and gH625 translocation, an accurate determination of the kinetic rate constants and of the association and dissociation processes, provides a broader understanding of its mechanism of translocation. The SPR results showed that the gH625-membrane interaction is a fast process that can be described by a two-step model initiated by peptide adsorption, primarily governed by electrostatic attractions, and followed by peptide insertion in the hydrophobic membrane core. We propose that after insertion in the hydrophobic core, the properties of the cell membrane promote peptide aggregation, translocation and cellular uptake.

However, aggregation does not modify the interaction of gH625 with lipids. This shows that oligomerization is not the consequence of the modified interaction of peptide with lipid bilayer but is the result of other processes. The presence of negatively charged phospholipids in the membrane improves the peptide/lipid monolayer interaction for gH625-H7 and gH625-G7 and decreases the interaction in the case of gH625. This indicates the importance of electrostatic interactions in binding to the membrane and of hydrophobic interactions in the crossing of the bilayer and also shows differences in affinity for various types of lipids. This selectivity could explain the ability of gH625 to cluster lipids in membrane bilayers and together with the shown propensity for the destabilization of the membrane it could contribute to the cell-penetration ability.

The membrane destabilizing and permeabilizing abilities of gH625 were also determined by observing GUVs under phase contrast microscopy, under fluorescence light sheet microscopy and by measuring their ability to induce leakage in a lipid bilayer, using LUV and GUV membrane mimetic systems. It was shown that both tested peptides do not destabilize GUVs, as demonstrated by the fact that the halo is retained along the observation. At the same time, we observe the presence of protuberances and an increase of the surface area, which can be correlated to the fusion of GUVs and to the intercalation and/or translocation of the peptide. Our data showed that both peptides induce an increase in the size of GUVs and also an increase of the number of larger GUVs, thus suggesting that gH625, and more markedly gH625-H7, is able to aid membrane fusion without destroying the GUVs’ structure; this is also supported by the fluorescence microscopy analysis, which showed higher fluorescence values for peptide-treated GUVs and FRET results which demonstrated that GUV fusion occurs up to few seconds from the administration of peptides. For the cell-internalization of CPPs, the destabilization of the membrane could be of relevance since it could provide facilitated passage of CPP and cargo through the membrane. Additionally, it could help the membrane to reorganize which is an important step in different mechanisms of escape from endosomes. The results that GUVs are not losing their halo effect, confirm that all the compounds (comprising also gH625-H7) do not produce leakage effects and hence pore formation which seem to be the cause of their reduced cell toxicity. In particular, even though in order to study multivalency we added positive charges, we are not switching from a translocation to an endocytic mechanism of internalization. Our results further support the view that multivalency and oligomerization enhance the uptake without modifying the mechanism of internalization.

It has been reported that positively charged peptides are able to induce membrane negative curvature which results in the formation of invaginations, while polymers produce positive curvature resulting in budding^51^. In our case, we observed formation of protuberances (positive curvature) which do not produce budding but seem to be reabsorbed by the GUVs which become larger and slightly loose circularity and roundness. Moreover, depletion of cholesterol increases polyarginine (R8) uptake independently from endocytosis^52^ possibly because of a transition from the liquid ordered to the liquid disordered arrangements which favours the interaction. In our case we believe that the further membrane rigidification, caused by the mainly hydrophobic peptides and showed by the calculation of membrane fluidity, could lead to lateral heterogeneity with regions of low tension in between different domains that may be exploited by the peptide to more easily perturb the membrane as also reported by others^35, 53, 54^.

Our study also covers the impact of the structural flexibility on the interaction of peptides with the membrane, as it was shown for several CPPs and related antimicrobial peptides (AMPs). It is well known that both types of these peptides are mainly non-structured in solution but they usually acquire at least in part α-helical or, in some cases, β-sheet structures. This is true also for gH625 for which it was shown that α-helical structure is associated with strong peptide– membrane interaction. Studies of gH625-H7 have revealed that dimerization promotes the formation of α-helical secondary structure and increases the interaction with lipid membrane. This is in accordance with our observations that oligomerization determines a stronger interaction with the membrane and might indicate greater inclination to fold in α-helix.

We have shown that oligomerization improves the insertion of gH625 into a lipid bilayer. The self-association (oligomerization) plays a key role in the process of membrane interaction and this has also been observed for antimicrobial peptides for which dimerization is known to control selectivity for specific targets and was previously proved also for other CPPs^55^. It is interesting to note that this result is in contrast with previous studies on TAT peptide, for which dimerization turned out to have no or little effect on translocation through cellular or model membranes^56–58^, and in fact, TAT is known to essentially employ the endocytic pathway.

The initial steps in peptide membrane interactions is induction of a higher degree of folding into a secondary structure; while surface bound, the peptide inserts into the lipid bilayer and the thermodynamics of this process seem to determine the actual uptake mechanism. Increasing evidences also indicate that membrane interacting peptides may exhibit cross-functionality. In fact, some AMP possess the ability to cross mammalian cell membranes by non-damaging processes, while several CPPs display significant antimicrobial activity^59–63^.

The α-helical AMPs could disrupt phospholipid membranes of bacterial cells by oligomerization of several peptide molecules, resulting in the death of target cells. A high degree of oligomer could be responsible of antibacterial activity while a lower degree could be necessary for membrane crossing. The main difference between multivalency and aggregation may be responsible for the different activities of membranotropic peptides which look apparently similar.

Moreover, we observe an absence of leakage, and hence pore formation, both in the LUVs and GUVs, which seems to be responsible of the lower toxicity of gH625 in comparison to other CPPs. The H7 moiety does not exert much influence on CPP interaction with a lipid membrane and it does not interfere with pore formation.

Some bacterial species are able to internalize into the intracellular compartment of mammalian cells and may cause persistent and/or recurrent infections due to inefficient intracellular killing of the bacteria^64^. Peptides able both to internalize into mammalian cell and kill bacterial pathogens might well act as potential candidates for establishing a novel treatment modality for intracellular infections. Further structural optimization might allow for future development of membranotropic peptides with antimicrobial activity and able to target internalized bacteria^5^.

In conclusion, multivalency is key for any design strategy which aims at targeting viruses, toxins, proteins, antibodies, and cell surfaces, as many of these biological targets have multiple repetitive binding sites. Multivalency stands out as a key principle for a targeted strengthening of any interaction between different interfaces correlated to the dramatically enhanced binding on a molecular scale. There are still a few reports analysing this subject in detail, which make a systematic study on models and complex systems and produce reliable predictions for the next chemical step. Multivalency is clearly advantageous because of its enormous binding strength and is increasingly being applied for pharmaceutically relevant targets^11, 65^. The first successful studies on multivalent drugs have begun to appear. However, this is a new drug concept which focusses on multivalency and holds a great potential in health applications.

## Methods

### Peptide synthesis

Peptides were prepared using the Fmoc-based solid-phase method as previously reported^6^, using a rink amide MBHA (0.54 mmol/g) resin and performing consecutive deprotection and coupling steps. Several cycles of deprotection (30% piperidine in dimethylformamide –DMF-, 5 min (2×)) and coupling (2.5 equivalents of amino acid +2.5 equivalents of 1-hydroxybenzotriazole (HOBT)/2-(1H-Benzotriazole-1-yl)-1,1,3,3-tetramethyluronium hexafluorophosphate (HBTU) (0.45 M in DMF) for 40 min) were performed. Side chain deprotection and cleavage from the resin were performed by treatment with a solution of trifluoroacetic acid (trifluoroacetic acid (TFA): H_2_O: thioanisole: ethanedithiol: anisole 85:5:5:3:2 v/v) for 4 h at room temperature. Following deprotection, peptides were precipitated in cold ethylic ether and analysis of the crudes was performed by LC–MS using a gradient of acetonitrile (0.1% TFA) in water (0.1% TFA) from 20 to 80% in 15 min. Purifications were performed by preparative RP-HPLC. All purified peptides were obtained with good yields (40–50%). Table 1 shows the sequences of all the synthesized peptides. Peptide oxidation was performed in ammonium bicarbonate 0.1 M pH 8.2 overnight. After oxidation the obtained compound was further purified by RP-HPLC. Peptide stock solutions were prepared in buffer with 2% dimethyl sulfoxide (DMSO).

### Preparation of unilamellar vesicles (small, large and giant)

LUVs and SUVs for fluorescence,circular dichroism and surface plasmon resonance, consisting of DOPG, DOPG/Chol (60/40 mol/mol), DOPC/Chol (60/40 mol/mol), and when necessary containing Rho-PE and NBD-PE, or ANTS and DPX, were prepared in 5 mM HEPES, 100 mM NaCl, pH 7.4^66^. Aliquots containing the necessary amount of lipids were dried from a chloroform solution with a nitrogen gas stream and lyophilized overnight to produce dry lipid films which were then resuspended in buffer and vortexed for 1 h; the lipid suspension was freeze-thawed 6 times to obtain multilamellar vesicles (MLV). LUVs with a mean diameter of 0.1 μm were prepared from MLV extruding the solution 10 times through polycarbonate membranes with 0.1 μm diameter pores. Lipid concentrations of liposome suspensions were determined by phosphate analysis^67^. SUVs were prepared from MLV by sonication for 30 minutes. GUVs consisting of DOPG/Chol (60/40) were prepared by gentle hydration method. The dry lipid mixtures (1 mg/ml) were hydrated overnight in 500 mM sucrose. The external buffer was removed by ultracentrifugation at 150.000 g/min, at 4 °C for 2 h, and the GUVs were resuspended in 500 mM glucose^68^.

### Membrane fluidity

Spectra were recorded 10 min after addition of peptides to LUVs (P/L molar ratio 0.05), using a 1 cm path length quartz cuvette, thermostated at 37 °C or 25 °C. All fluorescence spectra were corrected for the baseline signal. Laurdan emission spectra were recorded from 400 to 550 nm using a 365 nm excitation wavelength in the absence or presence of peptides in 5 mM HEPES, 100 Mm NaCl buffer (pH 7.4). The excitation generalized polarization (GP) was calculated as

3 $${\rm{GB}}=({{\rm{I}}}_{440}-{{\rm{I}}}_{490})/({{\rm{I}}}_{440}+{{\rm{I}}}_{490})$$

where I_440_ and I_490_ are the fluorescence intensities at the maximum emission wavelength in the ordered (440 nm) and disordered (490 nm) phases^69^.

### Lipid mixing and leakage measurements

Membrane fusion was monitored by the fluorescence resonance energy transfer assay (FRET)^70^ at 25 °C. Two populations of LUVs, one labelled with 0.6 mol% of NBD (donor) and Rho (acceptor) and one unlabelled were mixed and the variation in NBD fluorescence due to membrane fusion was determined. After the addition of aliquots of peptides the change in donor emission (excitation wavelength 465 nm, emission wavelength 530 nm) was monitored. In particular, labelled and unlabelled vesicles were mixed with a 1:4 ratio (final lipid concentration 0.1 mM) and small volumes (microliters) of peptides were added; the peptide was dissolved in dimethylsulfoxide (DMSO) (final concentration of DMSO in the peptide solution was always lower than 2%). The 100% of fusion was determined upon the addition of Triton X-100 (0.05% v/v). All results are an average of at least three experiments. A cut off filter at 515 nm was used.

To determine the peptide-induced mixing of the inner monolayer^71^, the NBD located on the outer monolayer of LUVs, was completely reduced by treatment with sodium dithionite (100 mM) for approximately 1 h on ice in the dark. The dithionite was removed through size exclusion chromatography, Sephadex G-50 DNA Grade, (GE Healthcare, Pharmacia, Uppsala, Sweden) with buffer containing 10 mM TRIS, 100 mM NaCl, and 1 mM EDTA, pH 7.4.

The ANTS/DPX assay^36^ was used to measure the ability of the peptide to induce leakage of the entrapped ANTS/DPX LUVs were prepared as described above, and 12.5 mM ANTS and 45 mM DPX were added to the lipid film and lyophilized; after hydration non encapsulated material was separated by gel filtration on a Sephadex G50 column. Increasing amounts of peptide dissolved in 5 mM HEPES and 100 mM NaCl at pH 7.4, were added to labelled vesicles (0.1 mM lipid) at 25 °C. The fluorescence intensities were recorded to quantify the leakage, the 100% leakage was calculated adding Triton X-100 (0.05% v/v).

### Tryptophan fluorescence measurements

Target vesicles (SUVs) were added to a peptide solution (4 μM) and the fluorescence intensity of the tryptophans (changes in the fluorescence intensity and blue shift of the wavelength maximum) was measured as a function of increasing amounts of SUVs, in three separate experiments. SUVs have been used to minimize light scattering effects^72^, excitation wavelength was set to 295 nm to avoid interferences from the tyrosine residue and spectra were recorded between 300 and 400 nm. Background fluorescence values from solvent and dilution factor were corrected. The lipid/peptide molar ratio was 200:1.

The association of peptides to lipid bilayers was determined experimentally as a partition equilibrium:

4 $${{\rm{X}}}_{{\rm{b}}}={{\rm{K}}}_{{\rm{p}}}{{\rm{C}}}_{{\rm{f}}}$$

where K_p_ is the apparent partition coefficient in units of M^−1^, X_b_ is the molar ratio of bound peptide per total lipid and C_f_ is the equilibrium concentration of the free peptide in solution as described elsewhere^6^. F_∞_ (which is the limiting fluorescence when all the peptide is lipid-bound) was extrapolated from the plateau region of the titration curve^41^. Knowing the fluorescence intensities of the free (F_0_) and bound (F_∞_, it was possible to calculate the fraction of membrane-bound peptide, fb, from the formula f_b_ = (F − F_0_)/(F_∞_ − F_0_) (equation 2), where F represents the fluorescence of the peptide at a given added lipid concentration. The equilibrium concentration of free peptide in solution, C_f_, and the extent of peptide binding X_b_ were obtained from f_b_. The peptides were initially partitioned only over the outer leaflet of the SUV (60% the total lipid)^40^; therefore, values of X_b_ were corrected as follows:

5 $${{\rm{X}}}_{b}^{\ast }={{\rm{X}}}_{{\rm{b}}}/0.6$$

The binding isotherms were obtained plotting X_b_^*^ versus C_f_. A straight line with the slope corresponding to K_p_ is the characteristic of simple partition equilibrium; however, deviations towards increased binding at higher peptide concentrations, are typical for cooperative binding of peptides able to self-associate at the membrane surface. In the latter case, the surface partition coefficient, K_p_, is estimated from the initial slopes of the curves.

Fluorescence quenching experiments were performed by adding acrylamide. A solution containing the peptide (4μM) in the absence or presence of LUVs was titrated with small aliquots of acrylamide (4 M); the maximal concentration of acrylamide used in this assay is 0.2 mmol/ml. The excitation and emission wavelengths were 295 and 340 nm, respectively^73^. The quenching constants (K_sv_), a measure of the accessibility of tryptophan to acrylamide, were obtained from the slope of the Stern-Volmer equation^74^; the equation is

6 $${{\rm{F}}}_{0}/{\rm{F}}=1+{{\rm{K}}}_{{\rm{sv}}}[{\rm{Q}}]$$

where F_0_ and F represent the fluorescence intensities in the absence and presence of the quencher (Q). As acrylamide does not significantly partition into the membrane bilayer^73^, K_sv_ is a good evidence of the bimolecular rate constant for collisional quenching of the tryptophan residue in the aqueous phase. Clearly, both the amount of non-vesicle-associated free peptide as well as the fraction of the peptide located at the bilayer surface contributes to K_sv_.

### Peptide aggregation

Peptide aggregation was monitored using ThioflavinT (ThT), which combines rapidly with aggregated peptides producing a new excitation maximum at 450 nm (slit width, 5 nm) and an enhanced emission at 482 nm (slit width, 5 nm)^43^. This experiment was conducted in LUVs (final phospholipid concentration of 0.1 mM) and buffer containing 25 μM ThT in 100 mM NaCl, 10 mM Tris–HCl, pH 7.4. Fluorescence was measured before and after the addition of peptides. In particular, we tested the following peptide concentrations 0.005, 0.01, 0.015, 0.02, 0.03, 0.04, 0.05 and 0.07 mM at 25 °C. Aggregation (A) was determined according to the following equation

7 $$ \% {\rm{A}}=({{\rm{F}}}_{{\rm{f}}}-{{\rm{F}}}_{0})/({{\rm{F}}}_{{\rm{\max }}}-{{\rm{F}}}_{0})\,\times \,100$$

where F_f_ is the value of fluorescence after peptide addition, F_0_ the initial fluorescence in the absence of peptide and F_max_ is the fluorescence maximum obtained immediately after peptide addition. Kinetic data were obtained at a concentration of 4 μM.

### Dynamic light scattering (DLS)

Particle size analysis was performed using Zetasizer Nano-ZS (Malvern Instruments, Worcestershire, UK). All measurements were performed at 25 °C in presence of LUVs composed by DOPG/Chol (60/40). To get insight into the influence of the peptides on LUVs integrity and size, LUV solution was analysed by DLS; the measurements were also conducted immediately after the addition of increasing concentrations of peptide.

### Binding Analysis by Surface Plasmon Resonance (SPR)

We used a BIAcore 3000 analytical system (Biacore, Uppsala, Sweden) equipped with a L1 sensor chip which contains hydrophobic alkanethiol chains, with exposed polar head groups; a lipid bilayer was created by introducing liposomes to the chip. SUVs composed by DOPG/Chol (60/40) (80 μL, 0.5 mM) were applied to the chip surface at a flow rate of 2 μL/min. NaOH 10 mM was used to remove any multilamellar structures from the lipid surface, and the flow rate was increased to 50 μL/min, which resulted in a stable baseline corresponding to the lipid bilayer linked to the chip surface. BSA was used as a negative control (25 μL, 50 μL/min 0.1 mg/μL in PBS) to confirm complete coverage of the nonspecific binding sites. Several sensorgrams were obtained with 30 μL of the peptide solutions injected at a flow rate of 5 μL/min, followed by PBS for 15 min to allow peptide dissociation. The plot at each peptide concentration of the SPR angle against time yields a series of sensorgrams which were fitted using numerical integration analysis (BIAevaluation software)^75^. The best fit was obtained by simultaneously fitting the sensorgrams with the two-state reaction model, which we also previously used to describe the binding mechanism of gH625^7^. The two states were hypothesized to correspond to the initial binding of the peptide (P) to lipids (L) to give PL followed by the formation of PL*, which may correspond to partial insertion of the peptide into the lipid bilayer.

8 $$P+L\,\leftrightarrow PL\leftrightarrow P{L}^{\ast }$$

The differential rate equations for this reaction model are reported, where RU_1_ and RU_2_ are the response units for the first and second steps, respectively; C_A_ is the peptide concentration; RU_max_ is the maximum peptide binding capacity (or equilibrium binding response); and k_a1_, k_d1_, k_a2_, and k_d2_ are the association and dissociation rate constants for the first and second steps, respectively.

9 $$dRU1/dt=ka1\times CA\times (RU\,{\max }\,-RU1-RU2)-kd1\times RU1-ka2\times RU1+kd2\times RU2$$

10 $$dRU2/dt=ka2\times RU1-kd2\times RU2$$

Kinetic data were assessed through the χ^2^ values, plots of the residuals from the model fitting and standard deviations. The quality of the fit to a specific parameter was deemed significant if the standard deviation was less than 10%.

### Circular dichroism spectroscopy

CD spectra were recorded in a 1.0 or 0.1 cm quartz cell at room temperature using a Jasco J-715 spectropolarimeter. The spectra are an average of 3 consecutive scans from 260 to 195 nm, recorded with a band width of 3 nm, a time constant of 16 s, and a scan rate of 10 nm/min. Spectra were recorded and corrected for the background contributions by substracting blank samples. Mean residue ellipticities (MRE) were calculated using the expression MRE = Obsd/(lcn), where Obsd is the ellipticity measured in millidegrees, l is the path length of the cell in cm, c is the peptide concentration in mol/l, and n is the number of amino acid residues in the peptide. The peptide concentration was 8 μM both in buffer (sodium phosphate 5 mM pH 7.4) and at different percentages of TFE. Peptide samples in DOPG/Chol (60/40 mol/mol) were prepared using the following protocol^32^: all peptides were first dissolved in TFE; immediately after preparation, the peptide solution was added to an equal volume of a chloroform/methanol solution containing the appropriate lipid concentration; solutions were dried with a nitrogen gas stream and lyophilized overnight; the dry samples were rehydrated with buffer to yield a final peptide concentration of 8 μM with a P/L ratio of 0.05 and extruded. The percentage of helix was calculated from measurements of their mean residue ellipticity at 222 nm^47^. We used [ϑ]222 values of 0 and −40.000(1–2.5/n) deg cm^2^ dmol^−1^ per amino acid residue for 0% and 100% helicity; n is the number of amino acid residues.

### GUV image analysis and evaluation of GUVs area

GUVs were used upon one day from preparation. Ten microliters of fluorescent GUVs were analyzed (before and after the addition of gH625 or gH625-H7) under a custom made light sheet fluorescent microscope equipped with five laser lines (*λ* = 375 nm Coherent Cube, *λ* = 405 nm Coherent Cube, *λ* = 473 nm Laserworld Blue-200, *λ* = 532 nm Coherent Compass 215 M-10 DPSS laser and *λ* = 632.8 nm Thorlabs HRR005S Helium-Neon laser), a motorized microscope MS-2000 XY stage with LX-4000 controller (Applied Scientific Instrumentation), a Focus Drive motorized Z stage (Tofra Inc.) and Nano-F200S nanopositioner controlled by Nanodrive® controller (Mad City Labs); all devices were managed by MicroManager software^76^. The illuminating objective was a Leitz LWD M10 with NA = 0.22 while the detection objective was a Leitz EF 25X with NA = 0,50. The light sheet was formed by a cylindrical lens f = 24.6 mm (Edmund Optics) mounted on a single mode optical fiber (Thorlabs) which receives the light from the illuminating objective and that is fixed to the detection objective through a modified objective coupled planar illumination system^77^. The emerging fluorescence was detected by the Orca flash 2.8 sCMOS camera (Hamamatsu) driven by Dalsa Xcelera (Teledyne DALSA) PCIe card mounted on AMD FX-8 core 3.5 GHz, 32 Gb RAM pc running Windows 8.1 (Microsoft). Eight-bit image stacks were calibrated using Rodbard NIH Image curve between 0 and 2.587, shadowed from west and background subtracted; finally, a threshold between 0.72 and 2.587 was used. GUV parameters were calculated by the Analyze particles ImageJ plugin; a minimum size of 5 μm^2^ and circularity of at least 0.10

11 $${\rm{Circularity}}=4\pi \times [{\rm{Area}}]/{[{\rm{Perimeter}}]}^{2}$$

with a value of 1.0 indicating a perfect circle) was considered. However, to avoid artifacts, areas below 10 μm^2^ were not considered for the total GUV number since values may not be accurate for very small particles. Light sheet fluorescent microscopy permitted to evaluate at least 2500 GUV per sample. All experiments were carried out in triplicate.

### Acceptor photobleaching fluorescence resonance energy transfer (FRET)

To evaluate the fusion of GUVs due to the addition of gH625 and gH625-H7, a slightly modified protocol for acceptor photobleaching FRET was performed. GUVs containing NBD and Rhodamine were mixed with no labelled GUVs in a 1:4 ratio. An aliquot of the mix was charged in the Burker’s chamber under Axioskop (Zeiss) fluorescence microscope; images were acquired using Axiocam Mrc5 CCD camera (Zeiss) in both at 525 nm and 615 nm and analysed with the Physiology module of Axiovision software 4.8 (Zeiss) to calculate the basic fluorescence of the donor. Samples were then treated with gH625 and gH625-H7 to a peptide:GUVs ratio of 0.5 and images were immediately acquired to calculate the fluorescence of the donor and the photobleaching of the acceptor (only used as a control for the amount of bleaching) after the exposure to peptides. At least 25 images were acquired and FRET signal evaluated by ImageJ 1.48 plug in “AccPbFRET”^78^. Furthermore, time series of fluorescence images were acquired before and after the addition of peptides (added at around 400 s) to the GUV suspension, to evaluate the dynamic of FRET (from 0 to 940 s). Fluorescence Images were then analysed with ImageJ 1.48 and integrated density (as the product of mean grey value of GUVs and their area) of approximatively 100 GUVs was calculated.

### Statistical analysis

Data are expressed as mean values ±SEM (±SD for integrated density calculation). Kruskal-Wallis one way ANOVA test with Dunn’s multiple comparison test was performed and differences were considered statistically significant when the P value was at least <0.05. All procedures were performed in triplicate.

## Electronic supplementary material

Supplementary Material Falanga et al

## References

[1] Ye, J. *et al*. CPP-Assisted Intracellular Drug Delivery, What Is Next? *Int J Mol Sci* **17**, doi:10.3390/ijms17111892 (2016). 10.3390/ijms17111892 PMC5133891 27854260

[2] Galdiero S (2012). Intracellular delivery: exploiting viral membranotropic peptides. Curr Drug Metab.

[3] Galdiero S (2014). Exploitation of viral properties for intracellular delivery. J Pept Sci.

[4] Falanga A, Cantisani M, Pedone C, Galdiero S (2009). Membrane fusion and fission: enveloped viruses. Protein Pept Lett.

[5] Galdiero S, Falanga A, Morelli G, Galdiero M (2015). gH625: a milestone in understanding the many roles of membranotropic peptides. Biochim Biophys Acta.

[6] Galdiero S (2010). The presence of a single N-terminal histidine residue enhances the fusogenic properties of a Membranotropic peptide derived from herpes simplex virus type 1 glycoprotein H. J Biol Chem.

[7] Galdiero S (2010). Role of membranotropic sequences from herpes simplex virus type I glycoproteins B and H in the fusion process. Biochim Biophys Acta.

[8] Falanga A (2012). Biophysical characterization and membrane interaction of the two fusion loops of glycoprotein B from herpes simplex type I virus. PLoS One.

[9] Galdiero S (2012). Structure and orientation of the gH625-644 membrane interacting region of herpes simplex virus type 1 in a membrane mimetic system. Biochemistry.

[10] Falanga A (2011). A peptide derived from herpes simplex virus type 1 glycoprotein H: membrane translocation and applications to the delivery of quantum dots. Nanomedicine.

[11] Guarnieri D (2013). Shuttle-mediated nanoparticle delivery to the blood-brain barrier. Small.

[12] Tarallo R (2011). Clickable functionalization of liposomes with the gH625 peptide from Herpes simplex virus type I for intracellular drug delivery. Chemistry.

[13] Borchmann DE (2015). Membranotropic peptide-functionalized poly(lactide)- graft -poly(ethylene glycol) brush copolymers for intracellular delivery. Macromolecules.

[14] Carberry TP (2012). Dendrimer functionalization with a membrane-interacting domain of herpes simplex virus type 1: towards intracellular delivery. Chemistry.

[15] Falanga A (2014). Elucidation of the interaction mechanism with liposomes of gH625-peptide functionalized dendrimers. PLoS One.

[16] Tarallo R (2013). Dendrimers functionalized with membrane-interacting peptides for viral inhibition. Int J Nanomedicine.

[17] Perillo E (2015). Quantitative and qualitative effect of gH625 on the nanoliposome-mediated delivery of mitoxantrone anticancer drug to HeLa cells. Int J Pharm.

[18] Perillo E (2016). Liposome armed with herpes virus-derived gH625 peptide to overcome doxorubicin resistance in lung adenocarcinoma cell lines. Oncotarget.

[19] Perillo E (2017). Synthesis and *in vitro* evaluation of fluorescent and magnetic nanoparticles functionalized with a cell penetrating peptide for cancer theranosis. J Colloid Interface Sci.

[20] Galdiero E (2017). Daphnia magna and Xenopus laevis as *in vivo* models to probe toxicity and uptake of quantum dots functionalized with gH625. Int J Nanomedicine.

[21] Valiante S (2015). Peptide gH625 enters into neuron and astrocyte cell lines and crosses the blood-brain barrier in rats. Int J Nanomedicine.

[22] Kaufman EA, Tarallo R, Falanga A, Galdiero S, Weck M (2017). Generation effect of Newkome dendrimers on cellular uptake. Polymer.

[23] Falanga A (2017). The intriguing journey of gH625-dendrimers. RSC Advances.

[24] Dinca A, Chien WM, Chin MT (2016). Intracellular delivery of proteins with cell-penetrating peptides for therapeutic uses in human disease. International Journal of Molecular Sciences.

[25] Lim SI, Lukianov CI, Champion JA (2017). Self-assembled protein nanocarrier for intracellular delivery of antibody. Journal of Controlled Release.

[26] Zhang D, Wang J, Xu D (2016). Cell-penetrating peptides as noninvasive transmembrane vectors for the development of novel multifunctional drug-delivery systems. Journal of Controlled Release.

[27] Han X, Tamm LK (2000). A host-guest system to study structure-function relationships of membrane fusion peptides. Proc Natl Acad Sci USA.

[28] Han X, Tamm LK (2000). pH-dependent self-association of influenza hemagglutinin fusion peptides in lipid bilayers. J Mol Biol.

[29] Badjic JD, Nelson A, Cantrill SJ, Turnbull WB, Stoddart JF (2005). Multivalency and cooperativity in supramolecular chemistry. Acc Chem Res.

[30] Fasting C (2012). Multivalency as a chemical organization and action principle. Angew Chem Int Ed Engl.

[31] von Krbek LK (2016). The Delicate Balance of Preorganisation and Adaptability in Multiply Bonded Host-Guest Complexes. Chemistry.

[32] Han X, Steinhauer DA, Wharton SA, Tamm LK (1999). Interaction of mutant influenza virus hemagglutinin fusion peptides with lipid bilayers: probing the role of hydrophobic residue size in the central region of the fusion peptide. Biochemistry.

[33] Reinhardt A, Neundorf I (2016). Design and Application of Antimicrobial Peptide Conjugates. Int J Mol Sci.

[34] Parasassi T, Conti F, Gratton E (1986). Time-resolved fluorescence emission spectra of Laurdan in phospholipid vesicles by multifrequency phase and modulation fluorometry. Cell Mol Biol.

[35] Maniti O, Alves I, Trugnan G, Ayala-Sanmartin J (2010). Distinct behaviour of the homeodomain derived cell penetrating peptide penetratin in interaction with different phospholipids. PLoS One.

[36] Parente RA, Nir S, Szoka FC (1990). Mechanism of leakage of phospholipid vesicle contents induced by the peptide GALA. Biochemistry.

[37] Thoren PE, Persson D, Lincoln P, Norden B (2005). Membrane destabilizing properties of cell-penetrating peptides. Biophys Chem.

[38] Galdiero S (2015). Antimicrobial peptides as an opportunity against bacterial diseases. Curr Med Chem.

[39] Yau WM, Wimley WC, Gawrisch K, White SH (1998). The preference of tryptophan for membrane interfaces. Biochemistry.

[40] Beschiaschvili G, Seelig J (1990). Melittin binding to mixed phosphatidylglycerol/phosphatidylcholine membranes. Biochemistry.

[41] Schwarz G, Stankowski S, Rizzo V (1986). Thermodynamic analysis of incorporation and aggregation in a membrane: application to the pore-forming peptide alamethicin. Biochim Biophys Acta.

[42] Rapaport D, Shai Y (1991). Interaction of fluorescently labeled pardaxin and its analogues with lipid bilayers. Journal of Biological Chemistry.

[43] Moreno MR, Pérez-Berná AJ, Guillén J, Villalaín J (2008). Biophysical characterization and membrane interaction of the most membranotropic region of the HIV-1 gp41 endodomain. Biochimica et Biophysica Acta (BBA) - Biomembranes.

[44] Andreetto E (2015). A Hot-Segment-Based Approach for the Design of Cross-Amyloid Interaction Surface Mimics as Inhibitors of Amyloid Self-Assembly. Angew Chem Int Ed Engl.

[45] Papo N, Shai Y (2003). Exploring Peptide Membrane Interaction Using Surface Plasmon Resonance:  Differentiation between Pore Formation versus Membrane Disruption by Lytic Peptides. Biochemistry.

[46] Shai Y (1999). Mechanism of the binding, insertion and destabilization of phospholipid bilayer membranes by alpha-helical antimicrobial and cell non-selective membrane-lytic peptides. Biochim Biophys Acta.

[47] Chakrabartty A, Kortemme T, Baldwin RL (1994). Helix propensities of the amino acids measured in alanine-based peptides without helix-stabilizing side-chain interactions. Protein Sci.

[48] Meng FG, Zeng X, Hong YK, Zhou HM (2001). Dissociation and unfolding of GCN4 leucine zipper in the presence of sodium dodecyl sulfate. Biochimie.

[49] Galdiero S (2008). Peptides containing membrane-interacting motifs inhibit herpes simplex virus type 1 infectivity. Peptides.

[50] Chetal P, Chauhan VS, Sahal D (2005). A Meccano set approach of joining trpzip a water soluble beta-hairpin peptide with a didehydrophenylalanine containing hydrophobic helical peptide. J Pept Res.

[51] Maniti O, Piao HR, Ayala-Sanmartin J (2014). Basic cell penetrating peptides induce plasma membrane positive curvature, lipid domain separation and protein redistribution. Int J Biochem Cell Biol.

[52] Fretz MM (2007). Temperature-, concentration- and cholesterol-dependent translocation of L- and D-octa-arginine across the plasma and nuclear membrane of CD34+ leukaemia cells. Biochem J.

[53] Lamaziere A (2008). The homeodomain derived peptide Penetratin induces curvature of fluid membrane domains. PLoS One.

[54] Epand RM, Epand RF (2009). Domains in bacterial membranes and the action of antimicrobial agents. Mol Biosyst.

[55] Hoyer J, Schatzschneider U, Schulz-Siegmund M, Neundorf I (2012). Dimerization of a cell-penetrating peptide leads to enhanced cellular uptake and drug delivery. Beilstein J Org Chem.

[56] Zhu WL, Shin SY (2009). Effects of dimerization of the cell-penetrating peptide Tat analog on antimicrobial activity and mechanism of bactericidal action. J Pept Sci.

[57] Chugh A, Eudes F (2007). Translocation and nuclear accumulation of monomer and dimer of HIV-1 Tat basic domain in triticale mesophyll protoplasts. Biochim Biophys Acta.

[58] Angeles-Boza AM, Erazo-Oliveras A, Lee YJ, Pellois JP (2010). Generation of endosomolytic reagents by branching of cell-penetrating peptides: tools for the delivery of bioactive compounds to live cells in cis or trans. Bioconjug Chem.

[59] Zhu WL, Hahm KS, Shin SY (2009). Cell selectivity and mechanism of action of short antimicrobial peptides designed from the cell-penetrating peptide Pep-1. J Pept Sci.

[60] Zhu WL (2006). Design and mechanism of action of a novel bacteria-selective antimicrobial peptide from the cell-penetrating peptide Pep-1. Biochem Biophys Res Commun.

[61] Nekhotiaeva N (2004). Cell entry and antimicrobial properties of eukaryotic cell-penetrating peptides. FASEB J.

[62] Splith K, Neundorf I (2011). Antimicrobial peptides with cell-penetrating peptide properties and vice versa. Eur Biophys J.

[63] Bahnsen JS, Franzyk H, Sandberg-Schaal A, Nielsen HM (2013). Antimicrobial and cell-penetrating properties of penetratin analogs: effect of sequence and secondary structure. Biochim Biophys Acta.

[64] Ciampolini J, Harding KG (2000). Pathophysiology of chronic bacterial osteomyelitis. Why do antibiotics fail so often? Postgrad Med J.

[65] Hild W (2010). G protein-coupled receptors function as logic gates for nanoparticle binding and cell uptake. Proceedings of the National Academy of Sciences.

[66] Hope MJ, Bally MB, Webb G, Cullis PR (1985). Production of large unilamellar vesicles by a rapid extrusion procedure: characterization of size distribution, trapped volume and ability to maintain a membrane potential. Biochim Biophys Acta.

[67] Fiske CH, Subbarow Y (1925). The colorimetric determination of phosphorus. Journal of Biological Chemistry.

[68] Morales-Penningston NF (2010). GUV preparation and imaging: minimizing artifacts. Biochim Biophys Acta.

[69] Amaro M, Reina F, Hof M, Eggeling C, Sezgin E (2017). Laurdan and Di-4-ANEPPDHQ probe different properties of the membrane. Journal of Physics D: Applied Physics.

[70] Struck DK, Hoekstra D, Pagano RE (1981). Use of resonance energy transfer to monitor membrane fusion. Biochemistry.

[71] Cummings JE, Vanderlick TK (2007). Aggregation and hemi-fusion of anionic vesicles induced by the antimicrobial peptide cryptdin-4. Biochim Biophys Acta.

[72] Ohki S (2006). Interaction of influenza virus fusion peptide with lipid membranes: effect of lysolipid. J Membr Biol.

[73] De Kroon AI, Soekarjo MW, De Gier J, De Kruijff B (1990). The role of charge and hydrophobicity in peptide-lipid interaction: a comparative study based on tryptophan fluorescence measurements combined with the use of aqueous and hydrophobic quenchers. Biochemistry.

[74] Eftink MR, Ghiron CA (1976). Exposure of tryptophanyl residues in proteins. Quantitative determination by fluorescence quenching studies. Biochemistry.

[75] Morton TA, Myszka DG, Chaiken IM (1995). Interpreting complex binding kinetics from optical biosensors: a comparison of analysis by linearization, the integrated rate equation, and numerical integration. Anal Biochem.

[76] Edelstein, A., Amodaj, N., Hoover, K., Vale, R. & Stuurman, N. Computer control of microscopes using microManager. *Curr Protoc Mol Biol* Chapter 14, Unit14 20, doi:10.1002/0471142727.mb1420s92 (2010). 10.1002/0471142727.mb1420s92 PMC3065365 20890901

[77] Holekamp TF, Turaga D, Holy TE (2008). Fast three-dimensional fluorescence imaging of activity in neural populations by objective-coupled planar illumination microscopy. Neuron.

[78] Roszik J, Szollosi J, Vereb G (2008). AccPbFRET: an ImageJ plugin for semi-automatic, fully corrected analysis of acceptor photobleaching FRET images. BMC Bioinformatics.

