# Supplementary material for: Dimerization in tailoring uptake efficacy of the HSV-1 derived membranotropic peptide gH625
Source: Sci Rep. 2017 Aug 25;7:9434. doi: 10.1038/s41598-017-09001-x (PMC5572722; doi:10.1038/s41598-017-09001-x)
Supplement: Supplementary file 1 — Supplementary Material Falanga et al [file 41598_2017_9001_MOESM1_ESM.pdf]

# Dimerization in tailoring uptake efficacy of the HSV-1 derived membranotropic peptide gH625.

Annarita Falanga,[1*] Salvatore Valiante,[2,3*] Emilia Galdiero,[2] Gianluigi Franci,[4] Olga Scudiero,[5] Giancarlo Morelli,[1] Stefania Galdiero[1*]

[1]Department of Pharmacy and CiRPEB- University of Naples "Federico II", Via Mezzocannone 16, 80134, Napoli, Italy

[2]Department of Biology, University of Naples "Federico II"– Monte Sant'Angelo, 80126, Napoli, Italy

[3]National Institute of Biostructures and Biosystems (INBB), V.le Medaglie d'Oro, 00136 Rome, Italy

[4]Department of Experimental Medicine, Università degli Studi della Campania Luigi Vanvitelli– Via De Crecchio, 80134, Napoli, Italy

[5]Department of Molecular Biology and Medical biotechnology, University of Naples "Federico II" 80131 Napoli, Italy and CEINGE-Biotecnologie Avanzate Scarl, Via G. Salvatore 486, 80145 Napoli, Italy

*These authors contributed equally to this work

***Address correspondence to:** Stefania Galdiero, Telephone: +39 081 2534503; Fax: +39 0812534560; Email: stefania.galdiero@unina.it

**Running head:** Multivalency role in gH625 uptake

**Dynamic light scattering (DLS).** Particle size analysis was performed using Zetasizer Nano-ZS (Malvern Instruments, Worcestershire, UK). All measurements were performed at 25 °C in presence of LUVs composed by DOPG/Chol (60/40). To get insight into the influence of the peptides on LUVs integrity and size, LUV solution was analysed by DLS; the measurements were also conducted immediately after the addition of increasing concentrations of peptide. The Table 1S reports the mean diameter (nm) and the polydispersity index (PDI) for the 3 peptides. Results clearly indicate that the PDI increases significantly with aggregation.

| **Table 1 S. Dynamic light scattering measurements** | | |
|---|---|---|
| **gH625** | | |
| **R=P/L** | **Mean Diameter(nm)** | **PDI** |
| **0** | 116.60±3.23 | 0.155±0.017 |
| **0.05** | 149.60±3.46 | 0.351±0.007 |
| **0.075** | 173.01±3.71 | 0.356±0.003 |
| **0.1** | 194.30±3.95 | 0.310±0.038 |
| **0.125** | 221.10±9.46 | 0.291±0.058 |
| **0.15** | 225.80±8.80 | 0.353±0.020 |
| **0.2** | 328.36±12.13 | 0.291±0.049 |
| **0.3** | 901.10±35.62 | 0.540±0.286 |
| **gH625-H7** | | |
| **0** | 118.30±1.92 | 0.157±0.018 |
| **0.005** | 179.80±3.14 | 0.322±0.007 |
| **0.01** | 336.4±29.36 | 0.346±0.034 |
| **0.015** | 609.50±62.64 | 0.459±0.019 |
| **gH625-G7** | | |
| **0** | 105.10±0.81 | 0.168±0.003 |
| **0.005** | 118.70±2.74 | 0.165±0.050 |
| **0.01** | 110.00±1.64 | 0.180±0.005 |
| **0.015** | 113.00±1.92 | 0.208±0.029 |
| **0.02** | 114.80±1.06 | 0.197±0.015 |
| **0.03** | 119.40±7.34 | 0.249±0.064 |
| **0.04** | 120.80±7.26 | 0.279±0.059 |
| **0.05** | 126.10±5.50 | 0.0272±0.034 |
| **0.07** | 150.10±9.12 | 0.246±0.021 |
| **0.1** | 308.40±30.23 | 0.376±0.016 |
| **0.2** | 1081.01±54.88 | 0.424±0.065 |

39
